# Basolateral protein Scribble binds phosphatase PP1 to establish a signaling network maintaining apicobasal polarity

**DOI:** 10.1016/j.jbc.2021.101289

**Published:** 2021-10-08

**Authors:** Regina B. Troyanovsky, Indrajyoti Indra, Rei Kato, Brian J. Mitchell, Sergey M. Troyanovsky

**Affiliations:** 1 Department of Dermatology, The Feinberg School of Medicine, Northwestern University, Chicago, Illinois, USA; 2 Department of Cell & Developmental Biology, The Feinberg School of Medicine, Chicago, Illinois, USA

**Keywords:** Scribble, phosphatase PP1, basolateral polarity proteins, apicobasal polarity, ABP, apicobasal polarity, BN-PAGE, blue native–polyacrylamide gel electrophoresis, GEF, guanine nucleotide exchange factor, LAP, leucine-rich repeat and PDZ domain, LRR, leucine-rich repeat, PP1, phosphatase 1, SBP, streptavidin-binding peptide, TJ, tight junction

## Abstract

Scribble, a member of the LAP protein family, contributes to the apicobasal polarity (ABP) of epithelial cells. The LAP-unique region of these proteins, which is essential and sufficient for ABP, includes a conserved Leucine-Rich Repeat (LRR) domain. The major binding partners of this region that could regulate ABP remain unknown. Here, using proteomics, native gel electrophoresis, and site-directed mutagenesis, we show that the concave surface of LRR domain in Scribble participates in three types of mutually exclusive interactions—(i) homodimerization, serving as an auto-inhibitory mechanism; (ii) interactions with a diverse set of polarity proteins, such as Llgl1, Llgl2, EPB41L2, and EPB41L5, which produce distinct multiprotein complexes; and (iii) a direct interaction with the protein phosphatase, PP1. Analogy with the complex between PP1 and LRR domain of SDS22, a well-studied PP1 regulator, suggests that the Scibble-PP1 complex stores a latent form of PP1 in the basolateral cell cortex. Such organization may generate a dynamic signaling network wherein PP1 could be dispatched from the complex with Scribble to particular protein ligands, achieving fast dephosphorylation kinetics.

The plasma membrane of most epithelial cells is divided into apical and basolateral domains. The apical domain faces the lumen, while the basolateral domain contacts adjacent cells and the extracellular matrix. These two membranes are separated by tight junctions (TJs) that seal the cells along the apex of their lateral surfaces. The group of Scribble proteins, identified by genetic screens in invertebrates, is responsible for basolateral membrane organization (1, 2, 3). This group in *Drosophila* consists of three proteins, Dlg, Lgl, and Scribble. In some cells, however, other proteins, such as Yrt, Cora, Na(+)/K(+) ATPase and integrins, can functionally compensate for the absence of Scribble proteins to maintain identity of the basolateral membrane (4, 5, 6). All of these basolateral identity determinants are evolutionarily conserved and also contribute to the polarity of mammalian epithelia. One of the functions of these proteins is to suppress the activity of the apical determinants, such as the Par6/aPKC complex, responsible for apical membrane organization. The mechanisms of how polarity determinants maintain corresponding membrane identities and how they communicate and suppress one another are not completely understood.

Scribble is a member of the Leucine-Rich Repeat And PDZ domain (LAP) protein family. The presence of four PDZ domains in Scribble suggests that this protein functions as a scaffold to generate specific macromolecular assemblies and localizes them to the specific subcellular sites. However, such Scribble-dependent assemblies that are essential for apicobasal polarity (ABP) have not yet been identified. Furthermore, only a small portion of Scribble (LAP Unique Region or sLUR) consisting of the Leucine-Rich Repeat (LRR) domain and two relatively short LAP-specific domains, LAPSDa and LAPSDb, is sufficient to maintain ABP (7, 8, 9). sLUR was reported to interact with two polarity proteins (recently reviewed in (10, 11)). It coimmunoprecipitates Lgl but not Dlg (12, 13, 14), and, by contrast, shows interaction with Dlg but not with Lgl in “in-cell” protein aggregation experiments (15). The relationship between these three proteins (Scribble, Dlg, and Lgl) appears to be cell-type-specific. In *Drosophila* follicle cells, the basolateral localization of Lgl but not Dlg depends on Scribble (16). In *Drosophila* midgut cells, by contrast, Scribble is needed for basolateral recruitment of both Dlg and Lgl (5).

The major function of scaffold proteins is to organize different enzymatic activities. Accordingly, one of the models suggests that Scribble and Dlg scaffold a phosphatase that protects Lgl from phosphorylation (16). Indeed, two phosphatases were shown to interact with sLUR. One of them is PHLPP1, which is reported to regulate AKT signaling but not ABP (17). The other is Protein Phosphatase 1 (PP1), which is proposed to interact with sLUR through the PP1-binding motif SILK (18, 19). However, major PP1-binding motifs of Scribble have been mapped to the PDZ domain region (18, 20). Furthermore, the SILK motif is embedded into a highly conserved β-sheet that forms the concave surface of the LRR domain of sLUR. Therefore, the SILK motif in sLUR is unlikely to be sufficient and unlikely to be available for interaction with PP1.

We recently showed that three LAP proteins, Scribble, Erbin, and Lano, play a critical and redundant role in mammalian ABP. Epithelial DLD1 cells are an established model for determining ABP mechanisms (14, 21) and, when deficient for these three proteins, show a phenotype that mimics Scribble loss in *Drosophila* epithelia, including abnormal distribution of TJs, cytosolic localization of both mammalian Lgl orthologs, Llgl1 and Llgl2 (Llgl1/2), and depolarized distribution of the Par6/aPKC complex. By contrast, Llgl1/2-deficient DLD1 cells show no strong ABP abnormalities, but they do show depolarization of Par6/aPKC complex (14). These observations suggest that in DLD1 cells, LAP proteins target several downstream effectors that are able to support ABP even upon deregulation of the Lgl-Par6/aPKC polarity pathway.

In order to determine such effectors, we have characterized sLUR-binding partners. We show that sLUR, its LRR domain in particular, participates in a broad spectrum of mutually exclusive interactions that include (i) sLUR dimerization, (ii) direct or indirect interactions with numerous mammalian orthologs of basolateral polarity proteins, including Lgl, Dlg, Yurt, Cora, and others, and (iii) interactions with two enzymes, GEF-H1, and all three isoforms of PP1—PP-1A, PP-1B, and PP-1G. Importantly, we find no evidence that sLUR scaffolds these proteins. Most likely, sLUR maintains PP1 in an inactive form at the particular location where it could be rapidly dispatched to its specific targets.

## Results

### sLUR forms homodimers

**Figure 1:**
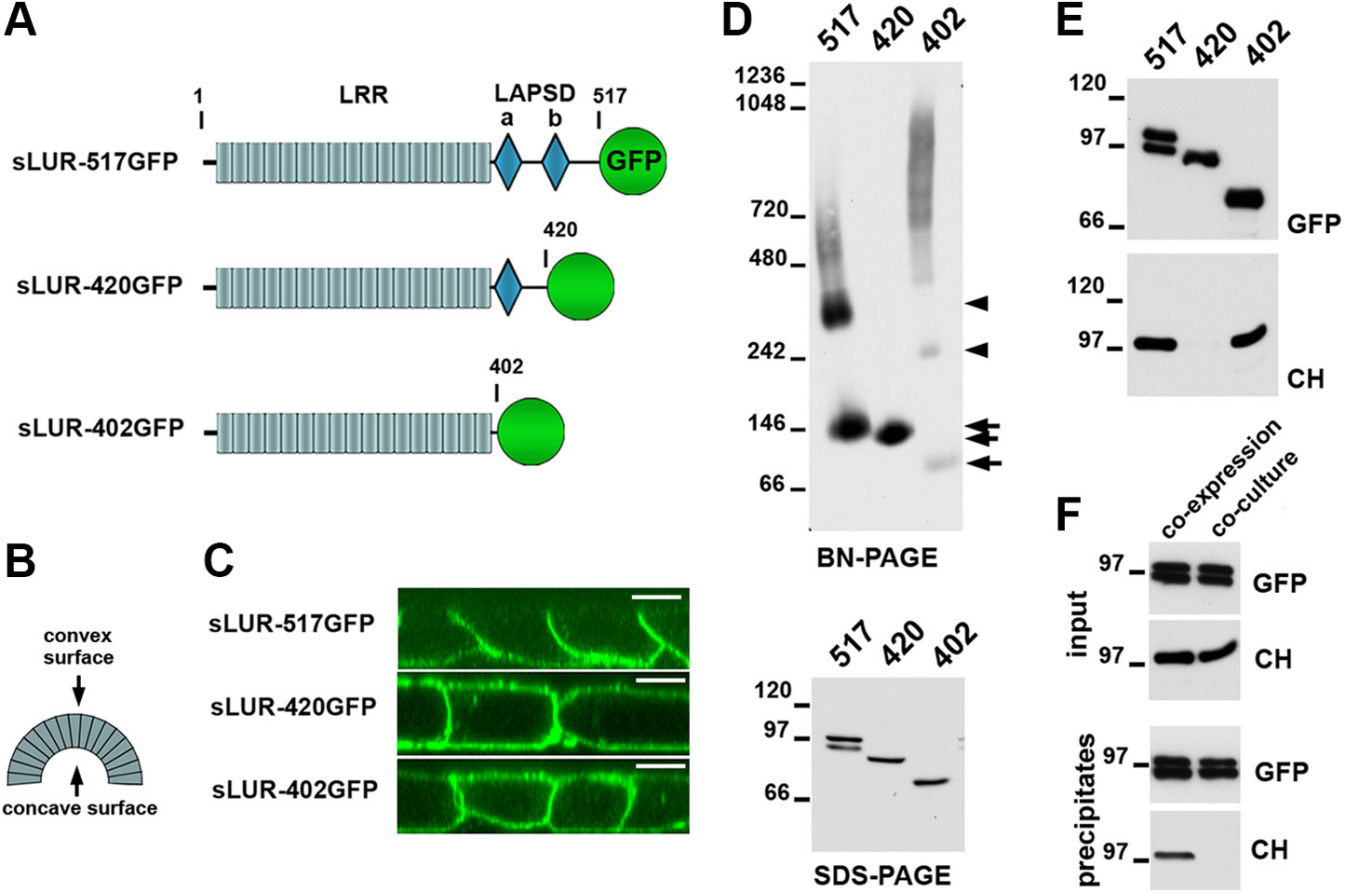
**Dimerization properties of sLUR mutants.***A*, schematic representation of sLUR-517GFP and two its mutants, sLUR-420GFP, and sLUR-402GFP, used in the study. The full-length sLUR (sLUR-517GFP) consists of a 17 LRR-long LRR domain (*gray*) and two LAPS domains, LAPSDa and LAPSDb (*blue diamonds*). The mutants lack LAPSDb (sLUR-420GFP) or both LAPSDa and LAPSDb (sLUR-402GFP). The number in the mutant name indicates position of their C-terminal residues. *B*, the LRR domains of all known LRR proteins form horseshoe-like structures creating convex and concave surfaces. In the majority of LRR proteins, the concave surface forms the ligand-binding sites. *C*, representative confocal optical sections of cells expressing the mutants shown in (*A*). Note that all mutants, except sLUR-517GFP, are localized at both apical and basolateral membranes. Bar, 10 μm. *D*, blue native gel electrophoresis (BN-PAGE) and conventional SDS-PAGE of the lysates obtained from cells expressing sLUR-517GFP (517), sLUR-429GFP (420), and sLUR-402GFP (402) probed for GFP. Note that BN-PAGE separates sLUR-517GFP in two major forms (∼150 kD and ∼300 kD) that correspond to the size of its monomer and its dimer. The array of additional minor faint diffuse bands was not specifically analyzed. The sLUR-420GFP cannot form a dimer, while sLUR-402GFP also exhibits a smear that suggests its extensive interactions with other proteins. *Arrows* and *arrowheads* indicate dimers and monomers, correspondingly. *E*, the cells expressing GFP-tagged mutants (indicated by numbers as in *C*) were cotransfected with sLUR-517mCH. Their lysates were then precipitated using GFP-trap and analyzed for GFP (GFP) and mCH (mCH) by Western blotting. Note that sLUR-420GFP, in contrast to other two mutants, is unable to coprecipitate sLUR-517mCH. *F*, the culture of cells coexpressing sLUR-517GFP and sLUR-517mCH and a coculture of cells separately expressing these two mutants were analyzed as in (*D*). Note that GFP-trap cannot coprecipitate the mCH-tagged form from the lysate obtained from the coculture. Data presented in *C* and *D* are representative of at least three independent experiments.

We have previously characterized three mutants of Scribble suitable for understanding its ABP function (Fig. 1, *A*–*C*, (14)). The full-length sLUR, sLUR-517GFP, fully rescues ABP upon expression in LAP protein-deficient DLD20-2 cells. The LAPSDb-deleted form of this mutant, sLUR-420GFP, is hyperactive, whereby it associates with both basolateral and apical cell membranes and provides them with some basolateral features. This basolateral “instructing” activity, but not the membrane-bound localization, is completely lost in the mutant sLUR-402GFP, which lacks both LAPSDa and LAPSDb. To determine what molecular characteristics could contribute to such dramatic functional differences of these three sLUR mutants, we compared them with Blue Native–Polyacrylamide Gel Electrophoresis (BN-PAGE). This technique identifies the most stable “core” protein complexes such as the cadherin-catenin complex or γ-secretase (22). This approach showed that the sLUR-517GFP runs as two major species ∼150 kD and ∼300 kD; the sLUR-420GFP ran exclusively as a low molecular, 140 kD form; and the sLUR-402GFP, in addition to the small amounts of 120kD and 240kD forms, was detected in a massive high molecular smear larger than 400 kD (Fig. 1*D*).

The lightest species of each mutant (150 kD, 140kD, and 120 kD, marked by arrows in Fig. 1*D*) approximately corresponded to the monomers, while the 300 kD and 240 kD species (marked by arrowheads) may be the homodimers. To test this assumption, we transfected the cells expressing the GFP-tagged mutants with the plasmid encoding mCH-tagged sLUR-517 (sLUR-517mCH) and analyzed the interactions between mCH- and GFP-tagged proteins using anti-GFP coimmunoprecipitation (Co-IP). This experiment confirmed that sLUR-517mCH formed a complex with sLUR-517GFP and with sLUR-402GFP, but not with sLUR-420GFP (Fig. 1*E*). The GFP- and mCH-tagged forms of sLUR-517 showed no coprecipitation in combined but separately prepared lysates (Fig. 1*F*). This control experiment demonstrates that the observed coprecipitation was not caused by aggregation of the mutants upon their solubilization. Altogether, these BN-PAGE results allow us to conclude that the 300 kD form is indeed the sLUR-517 homodimer. The fact that sLUR-402GFP coprecipitates sLUR-517mCH also suggests that this dimerization is mediated by the LRR domain. Finally, the loss of dimerization and functional “hyperacivity” of the LAPSDb-deficient mutant sLUR-420GFP suggest that dimerization plays a regulatory function.

### sLUR associates with all major basolateral polarity proteins

As an alternative strategy to identify sLUR protein interactions, we performed mass spectrometry (MS). BN-PAGE suggests that sLUR interactions, other than sLUR homodimerization, are weak and unlikely to be preserved in the standard Co-IP assay. To stabilize these weak interactions, we briefly cross-linked the cells using a homobifunctional cysteine-specific cross-linker, BMPEO3, with a 14.7 Å spacer arm before cell lysis.

**Figure 2:**
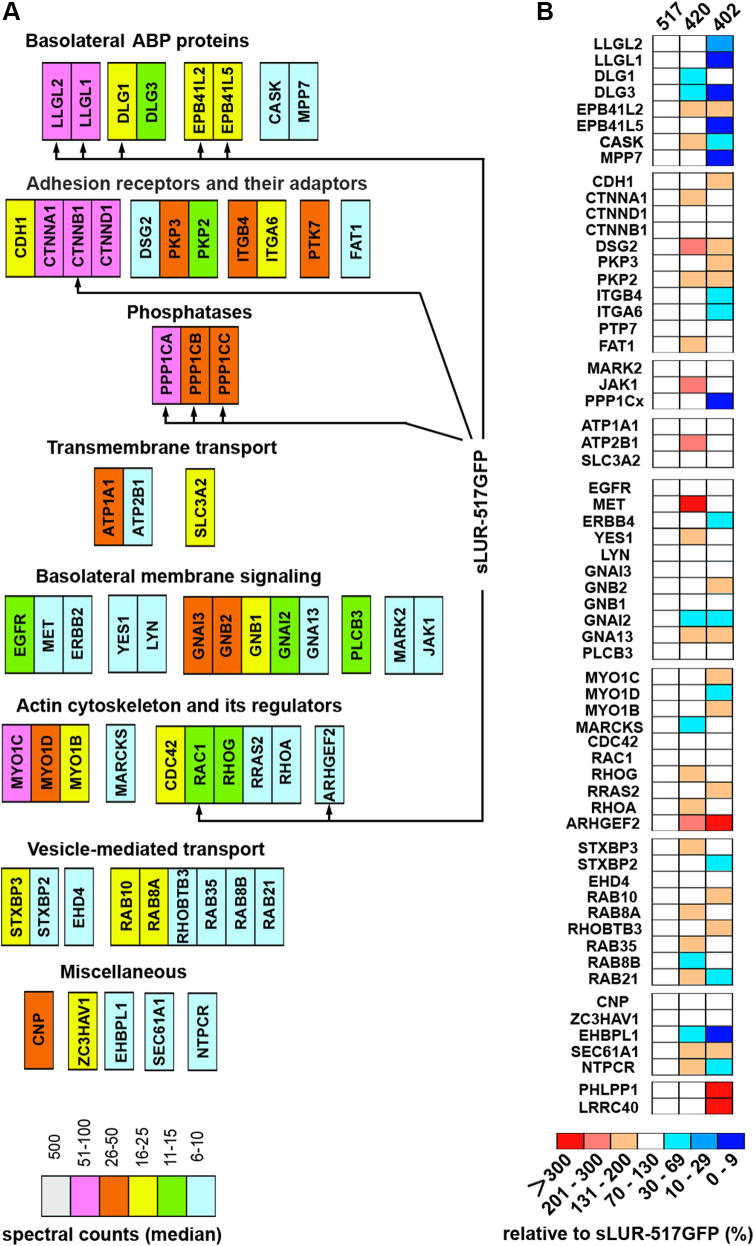
**Proteins associated with sLUR-517GFP and its mutants.***A*, diagram showing the median of spectral counts for each protein identified as associating with sLUR-517GFP in the cross-linking experiment. The proteins are named according to their gene symbols (see Table S1 for the exact values of spectral counts and for protein names). The identified proteins are classified in eight groups according to their function (indicated above each group). *Arrows* indicate proteins the presence of which in the GFP-trap precipitates was validated by Western blotting (see Fig. 3). *B*, changes in proteomes of sLUR-420GFP (420) and sLUR-402GFP (402) relative to that of sLUR-517GFP. The medians of spectral counts of each protein associated with the mutants are expressed in percent of the median values obtained for the control sLUR-517GFP proteome (see Table S1 for exact numbers).

The MS of the sLUR-517GFP precipitates obtained after this “in-cell” cross-linking experiment yielded about 500 proteins (see Tables S1 and S2). To select those with potential relevance to the ABP mechanism, we filtered our list through two control protein sets obtained by anti-GFP pull-down experiments using (i) wt DLD1 cells that discard nonspecific GFP-trap interactions and (ii) DLD20-2 cells expressing an inactive cytosolic sLUR mutant, sLUR-P305L-517GFP (14) that discards interactions with immature incorrectly localized sLUR (Table S2). The resulting protein list was additionally simplified by deletion of the low abundant proteins, represented by five or less spectra counts. The final tally consists of 64 proteins (Fig. 2*A* and Table S1) and was remarkably enriched with proteins reportedly involved in ABP regulation. All 64 proteins were subdivided into eight functional groups. The first group, “Basolateral ABP proteins,” includes proteins that have been mapped as basolateral determinants by Fly genetics: Dlg1 and Dlg3 (orthologs of Dlg), Llgl1 and Llgl2 (orthologs of Lgl), and EPB41L2 and EPB41L5 (orthologs of Cora and Yurt). This group also includes two other ABP proteins, CASK and MPP7. The most abundant proteins of the second group, “Adhesion receptors and their adaptors,” are members of the E-cadherin-catenin complex and α6β4 integrin. Cell–cell and cell–substrate adhesions mediated by these proteins have been proposed to be essential for ABP (23). Two other members of this group, FAT1 and PTK7, are essential components of planar cell polarity (24, 25). The group “Phosphatases” includes three isoforms of PP1 phosphatase (PP1): PPP1CA (PP-1A), PPP1CB (PP-1B), and PPP1CC (PP-1G). In total, the PP1 isoforms generated 128 spectra counts, more than any other protein in the interactome except sLUR itself (∼400 counts, see Table S1). The highlights of the next group, “Basolateral membrane signaling,” are three α subunits, GNAI3, GNAI2, and GNA13, and two β subunits, GNB1 and GNB2, of Guanine nucleotide-binding proteins (G proteins) as well as a protein kinase MARK2. These proteins have also been shown to participate in ABP in particular invertebrate models (6, 26). The proteins in the remaining functional groups are involved in “Transmembrane Transport,” “Actin Cytoskeleton and Its Regulation,” “Vesicle-mediated Transport” and “Miscellaneous.” Interestingly, one of the detected Actin and Cytoskeleton regulators, GEF-H1, could work as guanine nucleotide exchange factors (GEFs) for Rho during GNA13 activation (27, 28, 29).

### LAPSDa and LAPSDb independently contribute to the binding of sLUR to polarity proteins and PP1

We next compared the sLUR-517GFP proteome with that of the hyperactive sLUR-420GFP mutant and the inactive sLUR-402GFP mutant. The proteome of sLUR-420GFP was nearly identical to that of sLUR-517GFP (Fig. 2*B* and Table S1). By contrast, five of eight polarity proteins, Llgl1, Llgl2, Dlg3, EPB41L5, MPP7, as well as all three isoforms of PP1, significantly underperformed in the proteome of the nonfunctional mutant sLUR-402GFP. Remarkably, there was only one other protein, EHBP1L1, whose function is unknown, whose association with sLUR-402GFP was also dramatically reduced. In addition to the decreased representation of these seven proteins, the interactome of the sLUR-402GFP mutant showed a significant increase in GEF-H1, as well as the appearance of two new LRR-containing proteins, PHLPP1 phosphatase and LRRC40.

Taken together, our data shows that the fully functional sLUR-517GFP is able to homodimerize and to interact with a diverse array of basolateral polarity determinants. The hyperactive mutant sLUR-420GFP lost homodimerization properties, but fully retained the interactions with ABP proteins. By contrast, the nonfunctional mutant sLUR-402GFP retained homodimerization, but lost interactions with five key basolateral ABP proteins and with PP1. It also increased interactions with GEF-H1 and acquired the binding to PHLPP1 and LRRC40.

### The polarity proteins PP1 and GEF-H1 form distinct complexes with sLUR

**Figure 3:**
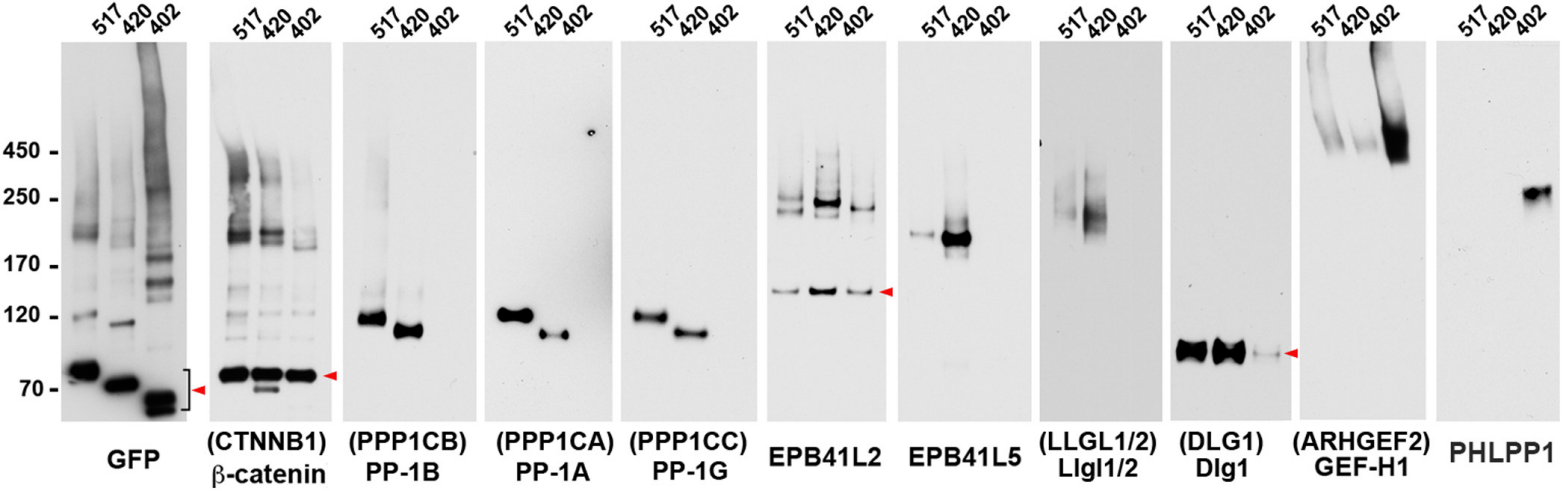
**Characterization of the BMPEO3 adducts of selected proteins associated with sLUR-517GFP and its mutants.** Cells expressing sLUR mutants, sLUR-517GFP (517), sLUR-420GFP (420), and sLUR402GFP (402) were cross-linked using BMPEO3, and their lysates were precipitated using GFP-trap as in the proteomics experiments. After SDS-PAGE, the precipitates were transferred on nitrocellulose and blotted with antibodies specific to GFP (GFP), β-catenin (β-catenin), PP1-B, PP1-A, PP1-G, EPB41L2, EPB41L5, Dlg1, GEF-H1, or PHLPP1. For consistency, the names of proteins are also provided as gene symbols (in *parenthesis*). Note that in all cases the adducts run as distinct bands. The monomeric form of each protein is indicated by *arrowheads*. Note also that the MWs of major adducts of all proteins decrease incrementally by about 10 kD upon LAPSDb deletion in sLUR-420GFP suggesting direct cross-linking of these proteins to the mutants. Data are representative of three independent experiments.

The GFP-trap precipitates obtained from BMPEO3-treated cells were also analyzed by Western blotting. The proteins for this analysis were selected based on their role in ABP and on antibody availability. This work confirmed that both sLUR-517GFP and sLUR-420GFP coprecipitate Dlg1, Llgl1/2 (Llgl1 and Llgl2), EPB41L2, EPB41L5, all three isoforms of PP1, GEF-H1, and β-catenin (Fig. 3). Also, consistent with MS analyses, Llgl1/2, EPB41L5, and all PP1 isoforms were undetectable in the sLUR-402GFP precipitate, which, by contrast, pulled down PHLPP1.

In addition to confirming the MS data, the SDS-PAGE analysis suggests how some of these proteins interact with sLUR. It showed that all three PP1 isoforms (30 kD) and EPB41L5 (88 kD) generated unique cross-linked adducts with sLUR517GFP (∼100 kD) at ∼130 kD and ∼200 kD, respectively. The size of these adducts decreased about 10 kD in the case of sLUR-420GFP. These adducts, therefore, approximate in their MW to heterodimers of the corresponding proteins. This result strongly suggests that PP1 and EPB41L5 form protein complexes with sLUR in which thiol groups involved in cross-linking, even considering protein flexibility (*e.g.*, (30, 31)), are ∼20 Å apart. The same is true in case of interactions between sLUR-402GFP (∼80 kD) and PHLPP1 (180 kD), which generated a unique cross-linked complex the size of which approximated their collective MW (∼250 kD). Two other proteins, Llgl1/2 (120 KD) and EPB41L2 (120 kD), also formed adducts approximated in MWs to their heterodimers with sLUR mutants (∼250 kD, in the case of sLUR-517GFP). However, these adducts appeared as several (two in case of EPB41L2) closely similar bands. Such multiplicity could result from the ambiguity of the Cys residues participating in the cross-linking reaction.

Another tested ABP protein, Dlg1, was coprecipitated only in a free form suggesting that the Dlg1-sLUR-containing complex had no cysteine pairs suitable for BMPEO3 cross-linking. Since we were unable to coprecipitate Dlg1 with any of the sLUR mutants without cross-linking (14), this result also suggests that BMPEO3 stabilized direct or indirect Dlg1-sLUR interactions by reinforcing the appropriate protein conformation or interactions with other proteins in the complex.

The proteins, which are significantly further away from one another in a protein complex than the length of the BMPEO3 spacer arm (14.7 Å), could be cross-linked only through intermediates. Such indirect cross-linking would increase both the size of the resulting adducts and the diversity of its composition. This is the case for GEF-H1. It formed an array of adducts with a MW of more than 450 KD, which is significantly larger than just the combined masses of GEF-H1 (∼110 kD) and sLUR (∼100 kD). Importantly, in agreement with proteomics data, GEF-H1-containing adducts were dramatically elevated in cells expressing the nonfunctional mutant, sLUR-402GFP.

Noteworthy, in few cases Western blotting did not exactly match the MS data. For example, Western blot (Fig. 3), but not MS (Fig. 2), showed that the amounts of the EPB41L5-and Llgl1/2-containing adducts were dramatically increased in sLUR-420GFP-expressing cells. Western blotting also showed a strong decrease of Dlg1 associated with sLUR-402GFP relative to other sLUR mutants. These discrepancies could be explained by the fact that Western blot detects adducts that migrate as specific bands. Such adducts most likely originate from specific complexes. In the case of spatial proximity without specific protein–protein interactions, the proteins would be expected to form a smear of adducts reflecting the absence of any specific alignments between the cross-linking proteins. The proteins in such smears would be detectable by MS but not by Western blot.

Overall, a combination of MS and Western blot data showed that sLUR-517GFP forms complexes with polarity determinants, Llgl, Dlg1, EPB41L2, EPB41L5, and with two enzymes, PP1 and GEF-H1. The functionally inactive mutant sLUR-402GFP is unable to interact with Llgl, EPB41L5, and PP1 and apparently with Dlg1, but interacts strongly with GEF-H1 and PHLPP1. By contrast, the hyperactive mutant sLUR-420GFP, which is unable to form homodimers, increases interactions with Llgl1/2, EPB41L5, and EPB41L2.

### Characterization of dimerization-incompetent sLUR mutants

**Figure 4:**
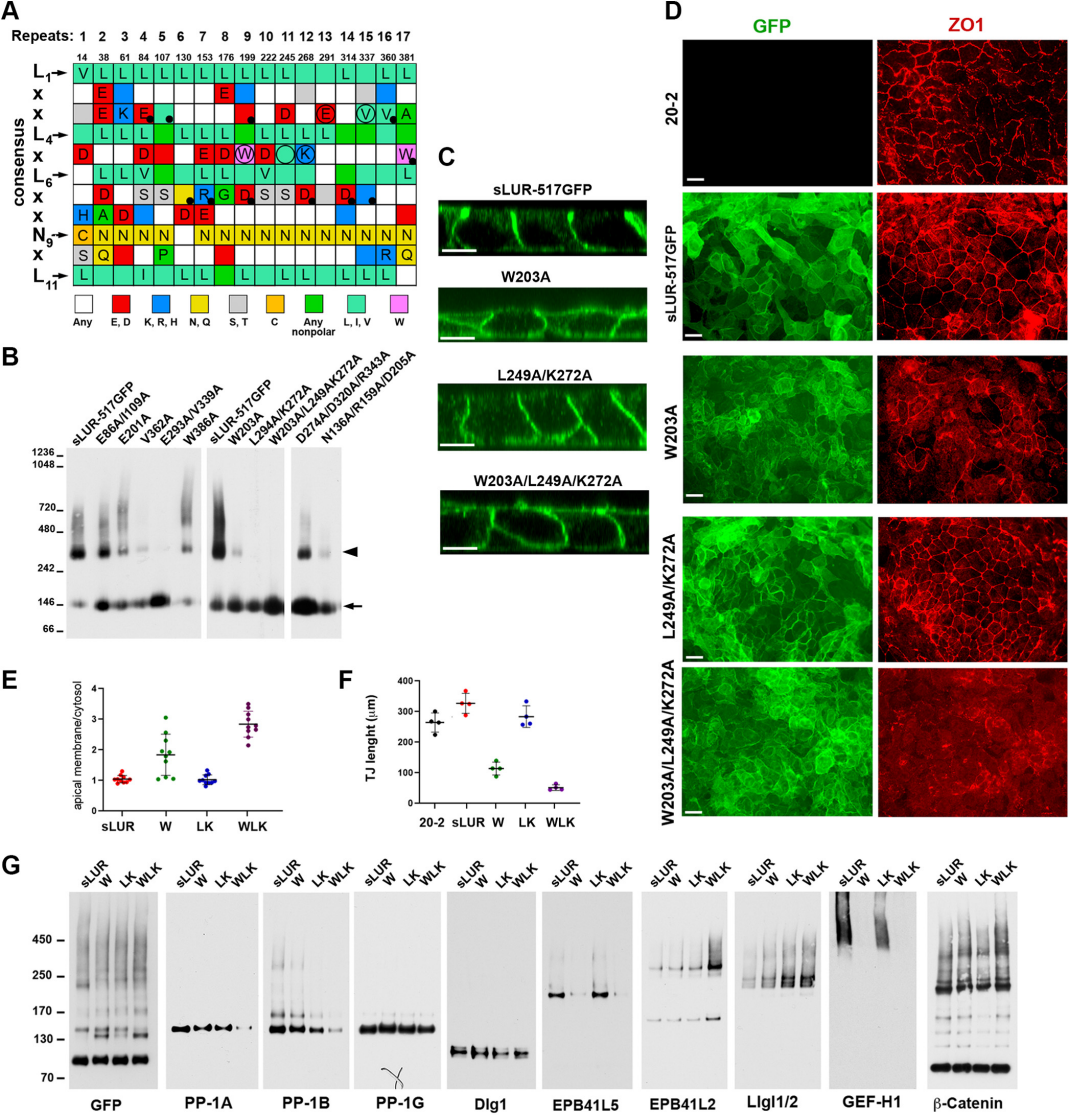
**Schematic representation and major characteristics of the sLUR point mutants.***A*, the conserved segments of 17 LRRs of Scribble are organized in numbered columns. These segments, with a consensus sequence LxxLxLxxNxL, form a β-sheet of the concave surface of Scribble LRR domain. Position of the first residue in each segment is indicated by a corresponding number. The consensus residues (*arrows*) in these segments form the core of the protein, whereas other residues are solvent-exposed. The residues are color-coded according to a presented chart. All residues that are identical in Scribble, Erbin, and Lano are marked by a single letter code. The residues tested by site-directed mutagenesis are marked by *dots*. The mutations that abolish dimerization are *circled*. *B*, BN-PAGE of the lysates obtained from DLD20-2 cells expressing control sLUR-517GFP (sLUR-517GFP) and its mutants (expressed by corresponding mutations). Data are representative of three independent experiments. Note that some of the mutations completely abolish formation of the high molecular weight dimeric form of sLUR-517GFP. *C*, representative confocal optical sections of LAP protein-deficient DLD20-2 cells (as a negative control), DLD20-2 cells expressing sLUR-517GFP (as positive control), and three of the dimerization incompetent mutants. Note that both mutants containing W203A mutations (W203A and triple mutation W203A/L249A/K272A) are localized at both apical and basolateral membranes. Bar, 10 μm. *D*, representative widefield images of these cultures showing GFP (*green*) and ZO1 (*red*) distribution. Note that expression of both mutants containing mutation W203A results in dramatic TJ disintegration. Bars, 20 μm. *E*, the ratio of the apical to cytosolic GFP fluorescence in cells depicted in (*C*). The measured apical areas do not include cell–cell contacts (n = 10). *F*, the continuous length of TJs in cultures shown in (*D*). Four images from two experiments were taken for each cell line. *G*, cross-linked adducts formed between the control protein, sLUR-517GFP (sLUR) and its mutants, sLUR-517GFP-W (W203A), sLUR-517GFP-LK (L249A/K272A), and sLUR-517GFP-WLK (W203A/L249A/K272A) and selected proteins. The equal amounts of protein samples obtained as indicated in Figure 3 were separated by SDS-PAGE and probed by Western blotting for GFP (GFP) that shows all adducts formed by the mutants, for three isoforms of PP1 (PP-1A, PP-1B, and PP-1G), for polarity proteins, Dlg1, EPB41L5, EPB41L2, Llgl1/2, as well as for β-catenin and GEF-H1. Data are representative of three independent experiments. Note that the triple sLUR-517GFP mutant, sLUR517GFP-WLK, exhibits no or weak interactions with PP-1A, PP-1B, EPB41L5, and GEF-H1, but its interactions with EPB41L2 and Llgl1/2 are stronger than in the case of the control protein. Also note that another dimerization-incompetent mutant, LK, forms nearly the same amounts of all adducts as the parental protein, sLUR-517GFP.

Comparison of sLUR-517GFP and sLUR-420GFP mutants suggests that sLUR homodimerization downregulates sLUR binding to some polarity proteins. To verify this, we sought to construct and characterize dimerization-incompetent sLUR mutants. Because the concave surface of the LRR domain is the most typical ligand-binding interface of LRR proteins (32) and because this surface can be predicted with high probability, we limited our mutagenesis to this area of sLUR. Indeed, almost all LRRs of sLUR start with an LRR invariant segment with the consensus sequence LxxLxLxxNxL (Fig. 4*A*), which is predicted to form an extended parallel β-sheet that defines the concave surface of the LRR domain (11, 32, 33). The conserved hydrophobic residues of this segment (“L”, at positions 1, 4, 6, 11) define the hydrophobic core, while conserved Asn or Cys (“N”) at position 9 (indicated by arrows in Fig. 4*A*) mediates interrepeat hydrogen bonds. The residues at positions 2, 3, 5, 7, 8 are exposed on the concave surface. Interestingly, a large number of these residues are conserved in all three LAP proteins, Scribble, Erbin, and Lano (Fig. 4*A*) supporting the idea that this surface is involved in protein–protein interactions essential for ABP. To test this idea, we constructed ten mutants by replacing individual or specific combinations of some of these conserved residues with Ala. The unchanged mobility of the mutants relative to the intact sLUR-517GFP in BN-PAGE (Fig. 4*B*) suggested that the incorporated mutations did not affect the protein folding or stability. BN-PAGE of these mutants also showed that the mutations of residues located in repeats 9 to 15 (circled in Fig. 4*A*) abolished sLUR homodimerization (Fig. 4*B*). By contrast, mutations in the repeats 3 to 7 showed weak or no effects. Three mutants, sLUR-W203A-GFP, sLUR-L249A/K272A-GFP, and sLUR-W203A/L249A/K272A-GFP (for simplicity abbreviated below as sLUR-W-GFP, sLUR-LK-GFP, and sLUR-WLK-GFP) were selected for more detailed characterization. The W203A mutation was selected because a bulky W203 residue is conserved in all LAP proteins and occupies a strategically important position at the very center of the concave surface. The mutants harboring mutation K273A (mutants sLUR-LK and sLUR-WLK) were selected because K273 is a part of a SILK motif, which has been suggested to contribute to the recruitment of PP1 into the complex with Scribble (18).

Fluorescence microscopy showed that all three tested mutants are localized at the cell cortex, which validates that the mutations we introduced had no effect on general sLUR structure. Interestingly, both mutants harboring mutation W203A lost exclusive basolateral localization and were also recruited to the apical cell cortex (Fig. 4, *C* and *E*). Furthermore, based on immunostaining for the TJ marker, ZO1, the cells expressing these two mutants, similar to cells expressing the dominant active mutant, sLUR-412GFP (14) exhibited severe defects in TJ formation (Fig. 4, *D* and *F*). Surprisingly, the sLUR-LK mutant, despite its dimerization defect, was very similar to the original sLUR-517GFP protein: it was exclusively localized at the basolateral cell cortex (Fig. 4*C*) and maintained the homogeneous honeycomb pattern of TJs in the transfected cells (Fig. 4, *D* and *F*).

### The binding with polarity proteins, PP1 and GEF-H1, is mediated by overlapping regions of the concave surface

Next, we studied whether selected mutations changed sLUR binding to proteins identified by our “in cell” cross-linking assay. Figure 4*G* shows that the W203A mutation completely abolished binding to GEF-H1 and significantly decreased binding to EPB41L5. Double mutation LK resulted in relatively minor changes in interactions in that it only slightly reduced binding to PP1-B but increased binding to Llgl1/2. The most severe consequences were detected for the WLK mutation in that the mutant nearly completely lost binding to PP-1A, PP-1B, EPB41L5, and GEF-H1 and, by contrast, increased binding to Llgl1/2 and EPB41L2. Remarkably, binding of the third isoform of PP1, PP-1G, to all three mutants remained unchanged. Taken together, these results strongly suggest that the concave surface of sLUR, in addition to the dimerization interface, encompasses partially overlapping binding sites to GEF-H1 and EPB41L5. The same surface also binds PP1, and furthermore these interactions showed some specificity for each PP1 isoform. Moreover, it may also contain the binding sites to Llgl1/2 and EPB41L2 such that the clear increase in binding of sLUR-WLK to both of these proteins could be explained by the lack of competition with other binding partners of the concave sLUR surface. Two proteins, Dlg1 and β-catenin, were unique since their binding to sLUR was unchanged upon our mutagenesis. These experiments demonstrated that the LK mutation is the most relevant one to study the functional role of sLUR dimerization because it minimally affected both the binding property of sLUR and its capacity to maintain TJ integrity.

### sLUR interactions with PP1 and polarity proteins are mutually exclusive

**Figure 5:**
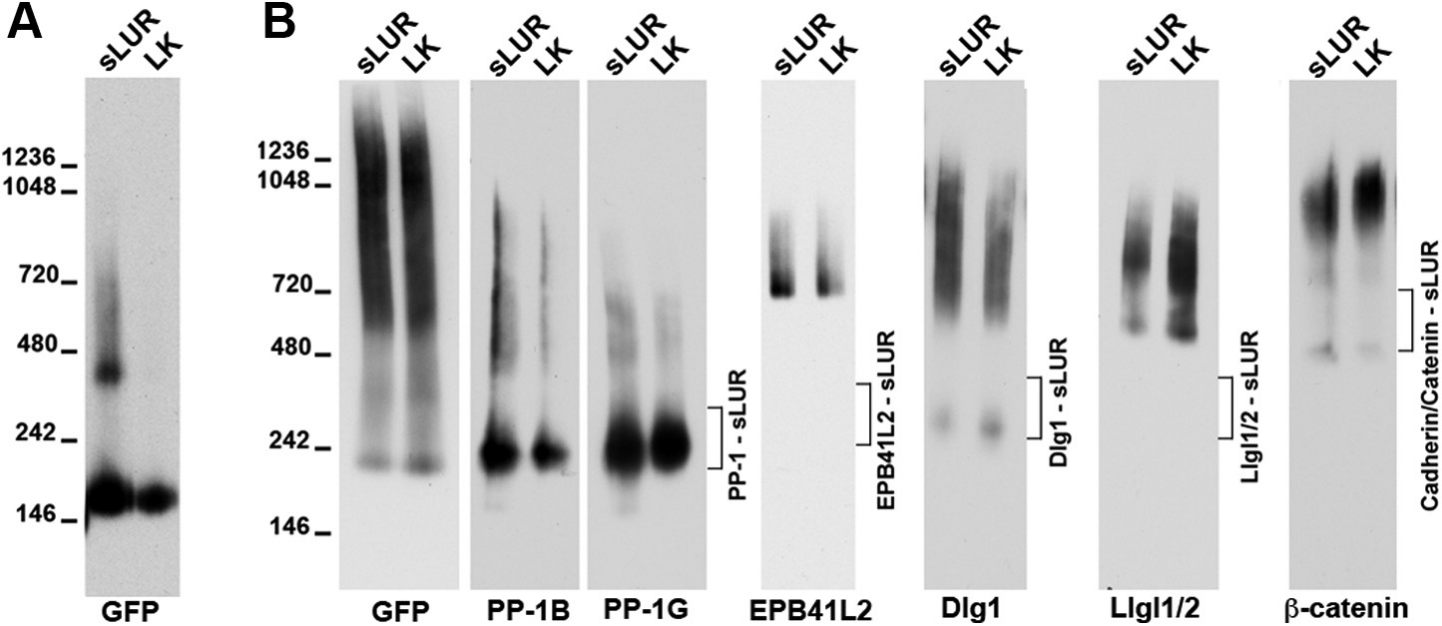
**Complexes between sLUR-517GFP and PP1 and polarity proteins are independent to sLUR dimerization.***A*, BN-PAGE of the lysates obtained from DLD20-2 cells expressing control sLUR-517GFP-SBP and its LK mutant. Note that the LK mutant cannot form dimers (*arrowhead*). *B*, the cells expressing sLUR-517GFP-SPB (sLUR) and its L249A/K272A mutant (LK) were cross-linked by BMPEO3 and then the complexes were isolated using Streptavidin-Agarose, eluted by Biotin in native conditions, and then processed by BN-PAGE. The gels were analyzed for PP1 (B and G isoforms), EPB41L2, Dlg1, Llgl1/2 and β-catenin. Data are representative of three independent experiments. The estimated migration of the complexes consisting just of sLUR and a given protein is indicated on the *right margins*. Anti-GFP staining showed that the sLUR complexes exhibit a broad variability of their MWs. The only complex detected as an isolated band corresponds to the sLUR-PP1 heterodimer. Note that the real sizes of all other complexes are significantly larger than estimated for the corresponding heterodimers.

The aforementioned cross-linking experiments suggest that sLUR dimerization and interactions with polarity proteins, such as Llgl1/2, EPB41L2, and EPB41L5, are mutually exclusive. To add more clarity to this important issue, we sought to determine whether the sLUR complexes with polarity proteins incorporate PP1. To this end, we expressed in DLD20-2 cells sLUR-517GFP and its sLUR-LK-GFP mutant, which had been additionally tagged with a short streptavidin-binding peptide (SBP). The SBP enables the elution of the tagged proteins from the streptavidin matrix in the native state (34). The BN-PAGE of sLUR double tagged with GFP and SBP tags retained dimerization, while we noted that the SBP tag, which is an unfolded stretch of residues, slightly reduced the level of dimers (compare Figs. 1*D* and 5*A*). As expected, the dimers were undetectable in case of sLUR-LK-GFP/SBP mutant (Fig. 5*A*). Next, the SBP-tagged complexes were precipitated from BMPEO3-treated cells and then eluted and analyzed using BN-PAGE. Anti-GFP staining of the gels revealed that both sLUR and its mutant migrated as a smear of MW greater than ∼200 kD that is consistent with cross-linking of both, sLUR and its mutant, to many proteins (Fig. 5*B*).

Strikingly, staining for PP-1B, PP-1G, Llgl, Dlg, and EPB41L2 (the proteins that retained their immunoreactivity to the antibodies in Western blotting after BN-PAGE) showed that MW of the corresponding complexes was independent of sLUR dimerization. It also showed that the complexes incorporating PP1 and polarity proteins were clearly different. The complexes with PP-1B and PP-1G migrated as relatively sharp band at ∼ 200 kD that corresponded to the sum of MWs of PP1 and monomeric sLUR. The MWs of complexes incorporating polarity proteins, by contrast, were approximately two times larger than estimated. For example, the complex incorporating EPB41L2 migrated at ∼ 700 kD instead of the estimated 300 kD. Another difference was that these complexes migrated as relatively broad bands that also suggested their complex organization. While additional work is needed to understand the exact structure of these complexes, their sizes in all cases did not match that of the sLUR-PP1 complexes.

### sLUR dimerization downregulates Scribble

**Figure 6:**
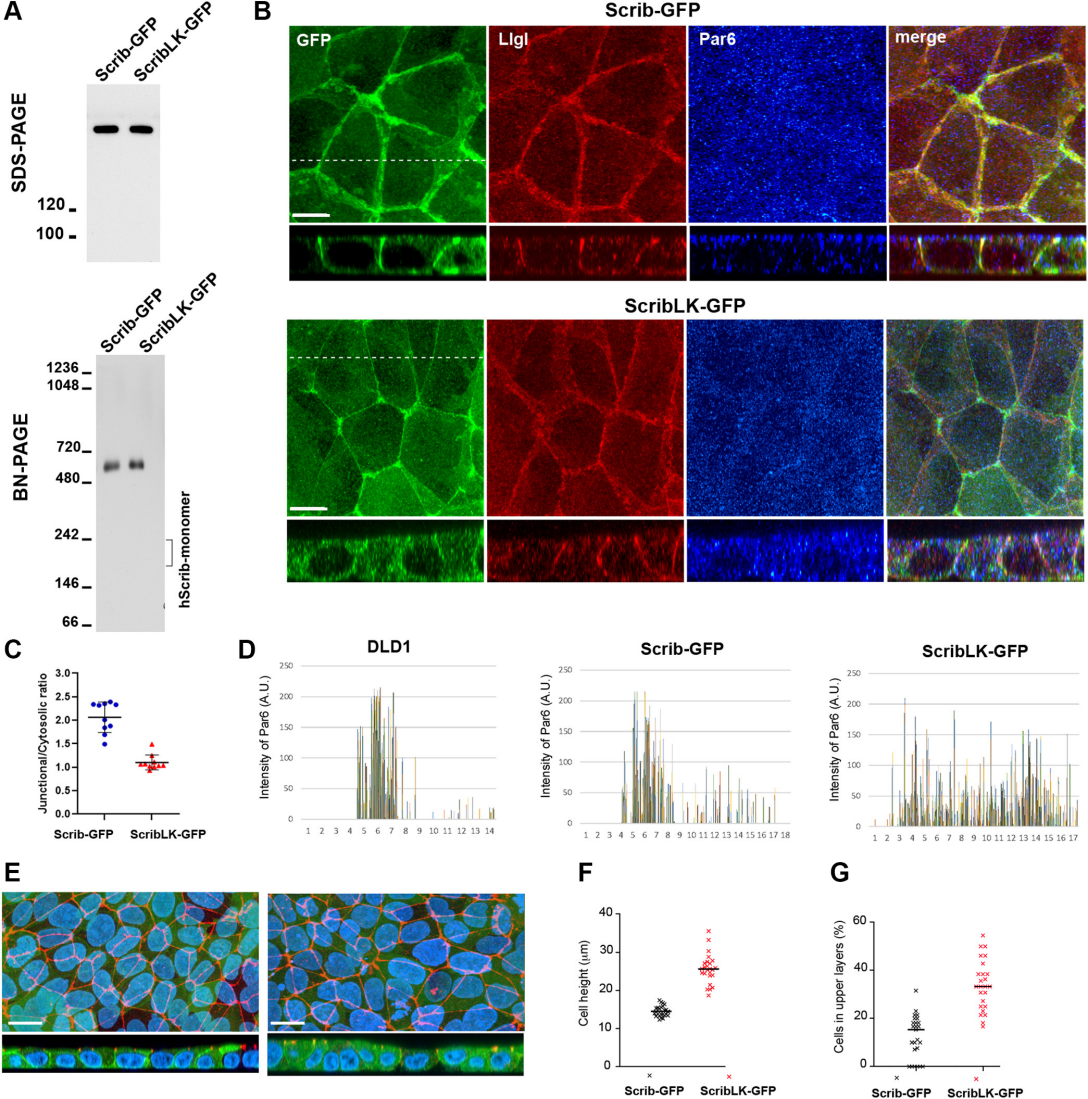
**Dimerization-incompetent mutant of sLUR enhances Scrib basolateral activity.***A*, SDS-PAGE and BN-PAGE of cell lysates obtained from DLD20-2 cells expressing GFP-tagged full-length hScrib (Scrib-GFP) or its L249A/K272A (ScribLK-GFP) mutant. Note that hScrib and its LK mutant exhibit the same MW in both types of PAGE. Data are representative of four independent experiments. *B*, projections of all x-y optical slices of DLD20-2 cells expressing Scrib-GFP (Scrib-GFP) and ScribLK-GFP mutant (ScribLK-GFP). The optical z-sections of the same magnification along the *dashed line* are shown at the *bottom*. Cells were imaged for GFP (GFP, *green*), basolateral marker Llgl1/2 (Llgl, *red*), and apical marker Par6 (Par6, *blue*). Bars, 10 μm. Data are representative of three independent experiments. *C*, concentration of the GFP-tagged mutants in the lateral cell cortex expressed as a ratio of peak fluorescence in the lateral cell cortex and the cytosol (n = 10). *D*, apicobasal plots of Par6 fluorescence intensity (*bars* of different colors represent intensities of individual groups of Par6-positive pixels) along the confocal slices (the step is 0.5 μm) derived from the full-view images presented in (*B*). The slice numbering starts from the most apical slice. The slightly different number of slices is due to variability in the cell heights. *E*, DLD20-2 cells expressing control Scrib-GFP and mutant ScribLR-GFP imaged for GFP (*green*), nuclei (DAPI, *blue*), and ZO1 (*red*). Data are representative of four independent experiments. Only merged images are shown. The optical z-sections through approximately the *middle* of each image are shown at the *bottom*. Bars, 20 μm. Note that both cell lines exhibit chicken-wire pattern of TJs. The height of the cells from the substrate to TJs (*F*) and number of the cells in the superficial layers (*G*) in these two cultures are assessed as described in Experimental procedures. The error bars represent SEs (n = 10). A.U., arbitrary unit.

Finally, by insertion of the LK mutation into the full-length human Scribble (hScrib) and by expression of the resulting mutant in DLD20-2 cells, we asked whether sLUR dimerization interface contributes to the function of this protein. SDS-PAGE showed that both Scrib-GFP and Scrib-LK-GFP were expressed at approximately the same level and run as a single sharp band (Fig. 6*A*). Surprisingly, BN-PAGE further showed that the LK mutation inserted into full-length hScrib had no effect on its MW in that both the intact and the mutant proteins run as a single species (∼500 kD), approximately twice the size of the monomeric protein (∼200 kD, Fig. 6*A*).

Then, we compared the DLD20-2 cells expressing Scrib-GFP and Scrib-LK-GFP for their ABP phenotype using an apical polarity marker, Par6, and a basolateral marker, Llgl1/2. As previously reported, the cells expressing the GFP-tagged intact hScrib, similar to the original DLD1 cells, exhibited correct localization of both markers—Par6 was predominantly at the apical cortex, while Llgl1/2 was clearly concentrated along the basolateral membrane (Fig. 6*B*). Additionally, we found that Scrib-GFP was localized along the entire basolateral cortex with very low levels of cytosolic fluorescence. Interestingly, this nearly exclusive basolateral localization was lost in case of the Scrib-LK-GFP mutant, which, in addition to basolateral staining, was also prominent in the cytosol (Fig. 6*C*). The apical marker Par6 was also predominantly cytosolic (Fig. 6*D*). By contrast, Llgl1/2 remained in the basolateral cortex.

While working with these cells, we noticed that the mutant expressing cells typically preserved their polarized epithelial appearance; however, they exhibited a tendency to form multilayered regions and were often much taller than their counterparts. To assess such differences, we costained the cells for TJs using ZO1 antibody, and for nuclei, using DAPI (Fig. 6*E*). This staining allowed us to assess two parameters—the average distance between cell substrate and the cell apex (Fig. 6*F*) and the frequency of regions with overlapped nuclei that reflects stratification (Fig. 6*G*). Three independent experiments clearly showed that despite the fact that mutant-expressing cells were able to form normal homogeneous chicken-wire pattern of TJs, they were indeed significantly taller and contained many more cells at the second layer.

## Discussion

The Scribble module proteins, Scribble, Lgl, and Dlg, are located at the basolateral cortex and contribute to the ABP of many epithelial cells (2, 3, 16). Here we addressed the molecular interactions of sLUR, the region of Scribble that is essential and sufficient to support ABP function. This region consists of an LRR domain and two short LAP protein-specific domains, LAPSDa and LAPSDb (10, 11). N-terminal nine residue-long segments of LRRs fold into an extended β-sheet, which paves a concave surface of the LRR domain. This concave surface of the LRR domain typically provides for the LRR domain proteins functionally important ligand-binding sites (32, 33). The proteins interacting with the concave surface of the LRR domains of LAP proteins have not been characterized.

Scribble, in cooperation with Dlg, is thought to protect Lgl from phosphorylation that keeps Lgl in association with the basolateral plasma membrane (16). This protection is lost at the apical membrane, where a Par6/aPKC complex phosphorylates Lgl. Many outstanding questions remain about this model, including how Scribble and Dlg prevent Lgl phosphorylation in the basolateral membrane and what molecular events underlie the Scribble-mediated “basolateral identity.” Scribble could be complimented by a large array of other basolateral proteins including E-cadherin, integrins, band 4.1 proteins (Yurt and Cora), sodium pump, G couple receptors and GEFs (5, 6, 23, 28, 35). Here we present evidence suggesting that the concave surface of sLUR directly or indirectly interacts with this web of polarity proteins and potentially controls their phosphorylation using protein phosphatase, PP1.

One of our important findings is that the sLUR concave surface, especially the area formed by LRRs 9 to 15, mediates formation of sLUR homodimers. It is the most straightforward explanation for many of our observations including one showing that mGFP- and mCherry-tagged forms of sLUR517 coprecipitate one another. This interaction is clearly the strongest among all sLUR-based interactions since it is the only one that is preserved in BN-PAGE. The hyperactive sLUR mutant, sLUR-420GFP, is unable to form homodimers that suggests that homodimerization is an “auto-inhibitory” mechanism deactivating sLUR activity responsible for the basolateral membrane identity. As clear evidence that such regulation exists, we show that the cells expressing a full-length hScrib mutant, harboring mutation L249A/K272A, which is the most specific toward preventing sLUR dimerization, “hyperdevelop” their lateral membrane. These cells are significantly taller than the control cells and show tendency to migrate into the superficial layers. These cells, despite “normal” appearance of TJs, also show clear abnormalities in localization of the apical determinant, Par6, suggesting that the hScrib L249A/K272A mutant has very high propensity to remove this protein from the cell cortex. This phenotype confirms our previous observation that Par6/aPKC signaling pathway is dispensable for ABP in DLD1 cells (14). BN-PAGE also showed that hScrib forms a 500 kD complex that potentially could be an hScrib homodimer since it is approximately twice larger than hScrib (200 kD). This complex could be organized by several independent dimerization hScrib interfaces, which keep the complex intact even in case of the L249A/K272 mutation. Interestingly, the nonfunctional sLUR mutant, sLUR-402GFP, exhibits strong interactions with two LRR domain proteins, PHLPP1 and LRRC40, which are undetectable in case of functional sLUR mutants. An attractive possibility is that the LRR domains of these two proteins form abnormal heterodimers with the LRR domain of sLUR-402GFP, thereby blocking the access of other targets to its concave surface.

To determine how sLUR could control basolateral identity of the cell membrane, we investigated its binding partners using “in-cell” cross-linking proteomics. In our approach, the representation of the ABP-relevant partners of sLUR was specifically enriched by filtering out the proteins that interact with the cytosolic sLUR mutant, sLUR-P305L-517GFP. This manipulation removes about 90% of all detected proteins, which apparently include nonspecific interactors and proteins that are involved in sLUR translation, maturation, and transport. Remarkably, the 64 remaining proteins include a broad set of polarity proteins that have been previously characterized by genetic screens in invertebrates. Among these proteins are mammalian orthologs of other members of the Scribble module, Dlg1, Dlg3, Llgl1, and Llgl2, as well as many proteins from alternative basolateral polarity pathways listed above, including E-cadherin and α6β4 integrin, their intracellular binding partners, mammalian orthologs of Cora (EPB41L2) and Yurt (EPB41L5), GEF-H1, G couple receptors, and many others. The most abundant protein of the obtained sLUR proteome is protein phosphatase PP1. Also, strikingly, the hyperactive mutant sLUR-420GFP that is unable to form dimers interacts with all of these proteins. By contrast, the nonfunctional sLUR mutant, sLUR-402GFP, which is located at the cell cortex as sLUR-420GFP, interacts with all of the same targets, except PP1, Lgl1/2, Dlg3, and EPB41L5. This observation suggests that this subgroup of proteins is important for Scribble to perform its role in ABP.

Our point mutagenesis mapped the sLUR binding sites for PP1, Lgl1/2, EPB41L5, and EPB41L2 to the same LRR concave surface that is involved in dimerization. This finding corroborates the idea that sLUR dimerization, which should enclose the concave surface, is a negative regulatory mechanism. The binding sites for these proteins obviously overlap but are not identical. Indeed, the binding to EPB41L5 is specifically reduced by the W203A mutation. By contrast, the binding to EPB41L2 and to Llgl1/2 is dramatically increased by the triple mutation W203A/L249A/K272A, which, at the same time, completely abolishes the binding to PP-1A and PP-1B. BN-PAGE of the cross-linked sLUR complexes provides additional evidence that these complexes are mutually exclusive. Indeed, our data strongly suggests that the sLUR complex with PP1 consists of only these two proteins. It is evident from the fact that the electrophoretic mobility of a sLUR-PP1 adduct, in both SDS-PAGE and BN-PAGE, exactly corresponds to the predicted dimeric sLUR-PP1 complex. Importantly, the sLUR complexes with Llgl1/2 and EPB41L2 in BN-PAGE are much larger than just a sum of corresponding proteins. However, all of our attempts to identify the simultaneous recruitment of two such proteins in these complexes were unsuccessful, leaving open the question about their exact structures.

The complex with PP1 appears to be the most abundant sLUR complex. PP1 is a versatile serine/threonine phosphatase, which binds to numerous ligands to target the resulting holoenzymes to specific locations and specific substrates. In most known cases, PP1-ligand binding is mediated by interactions between hydrophobic grooves on PP1 and short docking motifs of the ligands located at intrinsically disordered regions (36). Despite the fact that sLUR domain contains one such motif, SILK, it is unlikely that this accounts for the interaction between Scrib and PP1 as has been proposed (18): the SILK motif is not conserved in other LAP proteins, and it is embedded in the β-sheet and therefore likely not accessible. Consistent with this, our mutation of its K residue does not affect sLUR interaction with PP1.

A distinctly different mode of PP1 binding has been described for PP1 complex with the LRR domain protein SDS22. In this complex, PP1 interacts with the concave LRR domain surface of SDS22, and this interaction maintains PP1 in an inactive state (37, 38). It is proposed that the SDS22-PP1 complex provides PP1 for the rapid formation of new holoenzymes. Our data suggests that exactly this type of interaction mediates the binding of PP1 to sLUR. Firstly, the MW of the sLUR-PP1 complex and extremely efficient cross-linking of these two proteins suggest a direct interaction. Secondly, the combinatorial mutation W203A/L249A/K272A, but not a point mutation K272A within “SILK”, abolishes the sLUR binding to PP-1A and PP-1B. Thirdly, an evolutionary analysis shows a relationship between the LRR domains of LAP proteins and SDS22 (18).

**Figure 7:**
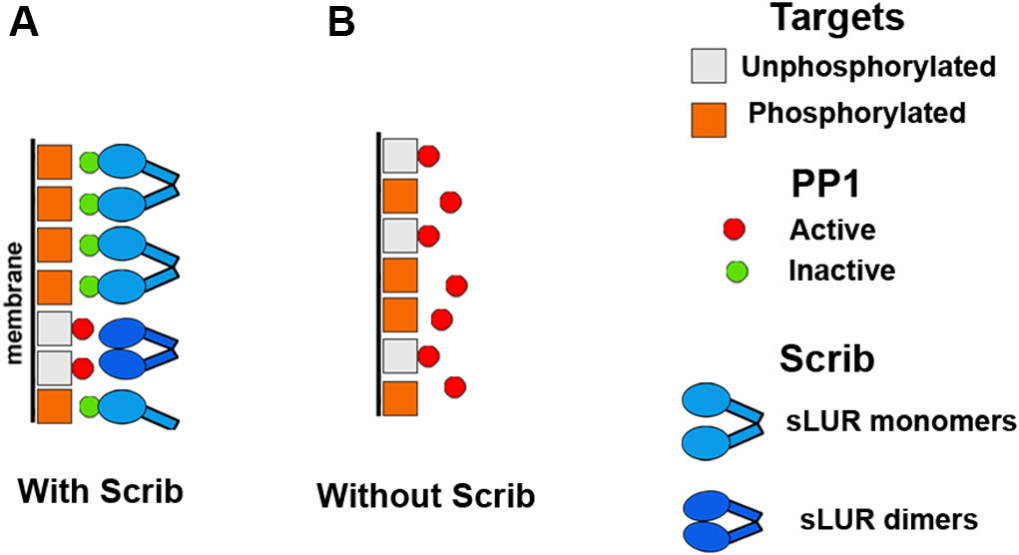
**Hypothetical model of the LAP protein signaling.***A*, Scribble (Scrib, *blue*) interacts with phosphatase PP1 keeping it in a short distance from the targets possibly in the inactive state (*green squares*) that preserves phosphorylated states of the nearby PP1 targets (*orange circles*). Dephosphorylation of the targets (*gray circles*) occurs only upon the release of the active PP1 (*red squares*) from the complex with Scribble. The PP1 release might be controlled by sLUR dimerization or by competitive binding of sLUR to the basolateral determinants, such as Dlg, Llgl or EPB41 proteins (not shown). By this mechanism, dephosphorylation reactions are linked to the specific sites and proceed with the fast kinetics. *B*, in case of Scribble deficiency, PP1 is constantly active. It results in chaotic dephosphorylation of targets without any spatial or kinetic specificity. It may also increase the level of dephosphorylated targets.

Taken together, the available data indicate that the concave surface of sLUR participates in three types of interactions. The first one is homodimerization that serves as an auto-inhibitory mechanism. The second one is interaction with the polarity determinants, Lgl1/2, EPB41L2, and EPB41L5. These interactions produce large protein complexes, the detailed structures of which remain to be identified. The third is a dimeric complex with PP1, which, by analogy with the SDS22-PP1 complex, may keep PP1 in an inactive state. Available data is not sufficient to place the complexes with Dlg1 to any of these groups. What role do these three types of complexes play in cell polarity? The most likely explanation is that Scribble and other LAP proteins provide a cortical pool of PP1 that is located in proximity to PP1 targets. Such cortical organization of PP1 generates a dynamic network in which PP1 could be immediately dispatched from the Scribble-PP1 complex to particular protein ligands (Fig. 7). The displacement of PP1 from the sLUR-PP1 complex may occur upon competitive binding of different polarity proteins to the concave surface of sLUR and/or to PP1. Accordingly, one of the major PP1-binding motifs, RVxF, is a signature of many polarity proteins, including Dlg and Lgl (36, 39). EPB41L5 also contains similar motif—RVKF (residues 119–122). Interestingly, SDS22 was also found to be involved in ABP (39, 40, 41, 42). This protein, however, is not a member of the basolateral group of proteins and rather resides at the apical cell cortex (42) suggesting that the SDS22-PP1 interactions might control formation of the apical cell domain.

Finally, we show that the sLUR concave surface also interacts with another enzyme, GEF-H1. It appears that GEF-H1 and sLUR form a large multiprotein complex since the cross-linked product containing both sLUR and GEF-H1 runs on SDS-PAGE as a broad band at a MW much larger than just a sum of two proteins. The point mutation W203A completely abolished this binding. While additional work is needed to determine the exact signaling pathway regulated by sLUR-bound GEF-H1, available data showed that the *Drosophila* GEF-H1 ortholog, Cyst, participates in ABP (28).

In conclusion, the protein–protein interactions of sLUR we revealed suggest a hypothesis that this part of Scribble participates in regulation of PP1 (and likely GEF-H1) within the basolateral cell cortex. The most attractive mechanism of this regulation is the formation of a basolateral layer of the LAP protein-bound inactive PP1 that is poised to be released to dephosphorylate particular targets, thereby increasing the kinetics and specificity of dephosphorylation. The release of the active PP1 from the complex with Scribble is apparently controlled by different polarity determinants that compete with PP1 for the same binding site located at the concave surface of sLUR. Availability of this site is also controlled by sLUR homodimerization. Since ABP could be maintained by LUR of other LAP proteins, such as Erbin and Lano (14), this mechanism could be universal for the entire family of these proteins and may contribute to a variety of signaling pathways that are based on protein phosphorylation.

## Experimental procedures

### Plasmids, cell culture, and transfection

The plasmids pRcCMV-sLUR517GFP and pRcCMV-ScribGFP were previously described (14). All point mutants described in the paper were generated using PCR-based site-directed mutagenesis by DNA Custom Cloning, Inc, and all plasmid inserts were completely sequenced before use. DLD1, LAP protein-deficient DLD20-2 cells, and DLD20-2 cells expressing sLUR-517GFP, sLUR-420GFP, sLUR-402GFP, sLUR-P305L-517GFP, and ScribGFP and their characteristics, including the expression levels of the recombinant proteins, have been described (14). Streptavidin-binding peptide (MDEKTTGWRGGHVVEGLAGELEQLR ARLEHHPQGQREP) inserted downstream to GFP in the sLUR-517GFP and sLUR-LK-517GFP was used for isolation of protein complexes. Transfection and growth of DLD1 cells and their progenitors were done as described (43). After antibiotic selection, the cells expressing GFP-tagged proteins were sorted for moderate transgene expression by FACS. The resulting positive cells were then cloned in order to obtain cells with desirable expression levels of the transgene. The levels and sizes of the recombinant proteins in the obtained clones were analyzed by Western blotting. At least three clones were selected for each construct and all were tested in most of the assays. All clones of cells expressing a particular transgene exhibited the same phenotype. A representative data for one of three clones is presented.

### Immunofluorescence microscopy

For wide-field immunofluorescence, cells were cultured on glass coverslips 48 to 64 h. The cells were fixed with 2% formaldehyde (10 min) and then permeabilized with 1% Triton X-100 (see (43) for details). The images were taken using Eclipse 80i Nikon microscope (Plan Apo 40×/1.0 for Fig. 4*D*) using a digital camera (CoolSNAP EZ; Photometrics, Tucson, AZ). For confocal microscopy, the cells were cultured 48 to 64 h on glass-bottom dishes (P35G-1.5; MatTek). Immediately before imaging, the dishes were filled with 97% glycerol. The images were taken using Nikon A1 laser scanning confocal microscope (Z step size was 0.5 μm in all cases) equipped with TIRF 100×/1.45NA objective lens (Figs. 4*C* and 6*B*) or Plan Apo 60×/1.4 Oil DIC N2 (Fig. 6*E*). The images were then processed using Nikon's NIS-Elements software.

The following antibodies were used: rabbit antibodies: anti-Scribble, anti-GEF-H1, anti-Llgl1/2, anti-PP-1B (ab36708, ab155785, ab18302, and ab53315; Abcam), anti-EPB41L2 (PA5-82257; Invitrogen); anti-PHLPP1 (22789; Proteintech) anti-EPB41L5 and mCherry (NBP2-38354 and NBP2-25157; Novus Biologicals); mouse antibodies: anti-Dlg1 and anti-β-catenin (610874 and 610154; BD Biosciences); anti-PP-1A, anti-PP-1G, anti-Llgl2, anti-ParD6B, anti-GFP (sc-271762, sc-515943, sc-376857, sc-166405, sc-9996, correspondingly; all Santa Cruz Biotechology). Specificity of all antibodies was tested using Western blotting and CRISPR/Cas9 (except PHLPP1) knockout. All secondary antibodies were purchased from Invitrogen. All images presented in the paper are representative of at least three independent experiments.

### Proteomics

The confluent cultures of indicated cells grown on 10 cm plates were cross-linked using BMPEO3 cross-linker (1 mg/ml in ice-cold PBS), then lysed with the Lysis Buffer (LB, 20 mM TrisHCl, 150 mM NaCl, 2 mM EDTA, and 1% Triton X-100), and cleared by centrifugation and incubated for 1 h with 30 μl of GFP-trap beads (Chromotek). After incubation, the beads were washed four times in LB, boiled in 30 μl of SDS-sample buffer, and loaded on SDS-PAGE. The samples were run through 4 to 12% SDS-PAGE, and the samples were submitted to the Proteomics facility of Northwestern University where they were subjected to in-gel reduction, alkylation, and finally in gel tryptic digestion, performed overnight by adding 2 μg trypsin at 37 °C. Peptides were analyzed by LC-MS/MS using a Dionex UltiMate 3000 Rapid Separation nanoLC coupled to an Orbitrap Elite Mass Spectrometer (Thermo Fisher Scientific Inc). Proteins were identified from the tandem mass spectra extracted by Xcalibur version 4.0. MS/MS spectra were searched against the SwissProt *Homo sapiens* database (version 2019.09, 20,432 entries) using Mascot search engine (Matrix Science, London, UK; version 2.5.1). All searches included carbamidomethyl Cys as a fixed modification and oxidized Met; deamidated Asn and Gln; and acetylated N-term as variable modifications. Three missed tryptic cleavages were allowed. The MS1 precursor mass tolerance was set to 10 ppm and the MS2 tolerance was set to 0.6 Da. The search result was visualized by Scaffold v 5.0.1 (Proteome Software, INC). A 1% false discovery rate (FDR) cutoff was applied at the protein level. Peptide FDR was calculated as the sum of the Exclusive Spectrum Counts of decoy proteins divided by the sum of the Exclusive Spectrum Counts of target proteins, converted to a percentage. Only proteins with a minimum of two unique peptides above the cutoff with 99% threshold were considered for further study.

A total of seven samples independently obtained from sLUR-517GFP-expressing cells were quantified. Using R Studio (Version: 3.6.0 [2019-04-26]), each sample column was joined by their respective unique ID (gene name) to reproduce a merged data frame. Proteins with less than or equal to five identifications across all seven samples were excluded from further data processing. Mean spectra counts were then calculated for the remaining proteins. The proteins with mean spectra counts below five were also discarded. Similar technique described above was applied to obtain maximum spectra count values for the samples obtained from DLD1 cells (three samples) and mean spectra counts for the samples obtained from sLUR-P305L-517GFP-expressing cells (three samples). These values were then applied against mean values of sLUR-517GFP to identify contaminants. In both combinations, proteins with spectra counts greater than 20% of sLUR-517GFP mean were subject to removal, with the exception of Scribble. The same procedure was used for analyses from 3 to 5 samples of sLUR-420GFP- and sLUR402GFP-expressing cells.

### BN-PAGE, SDS-PAGE, and immunoprecipitation

The BN-PAGE was performed according to the manufacturer's protocol (Native-PAGE Novex Bis-Tris Gel system; Invitrogen). In brief, a 3-day-old confluent culture in a 3-cm tissue culture dish was washed in phosphate-buffered saline (PBS) and cross-linked, if indicated, by incubation for 5-min with cysteine-specific cross-linker BMPEO3 (Thermo Fisher) at 4 °C as indicated above. The reaction was stopped by washing the cells with PBS with dithiothreitol (final concentration, 1 mM). The cells then were extracted in 0.5 ml of lysis buffer (50 mM Bis-Tris, pH 7.4, 50 mM NaCl, 1 mM EDTA, and 1% of Triton X-100). In some cases, 40 μM protease inhibitor 4-(2-aminoethyl)benzenesulfonyl fluoride (AEBSF) (Calbiochem) and phosphatase inhibitor cocktail (1 mM Na3VO4, 10 mM NaF, and 2 mM β-glycerophosphate) were added. After 10-min (14,000 rpm at 4 °C) centrifugation, the lysates were mixed 1:1 with gel loading buffer and placed on the BN-PAGE gel. The NativeMark Protein Standard (LC0725, Invitrogen) was used for MW estimation. The MW standards were loaded on each gel, and their positions were marked on the membrane (Immobilon-P, Millipore) after blotting. For biotin-based protein isolation, the cell lysates obtained as described above were incubated with streptavidin-agarose (Sigma) followed by elution with biotin (30). The eluted complexes were analyzed by BN-PAGE as indicated above.

For conventional Western blot analysis, the GFP-trap precipitates together with a sample of protein markers (10–460 kDa; HiMark #LC5699, Invitrogen) were separated on precast 3 to 8% Tris-acetate gels (Invitrogen), which are ideal for the separation of large MW proteins. After blotting, the proteins on nitrocellulose membrane (Millipore) were temporarily stained by 0.05% solution of Naphthol Blue Black (in 10% methanol, 5% Acetic Acid), and the positions of the MW standards were marked. The cross-linking and immunoprecipitation were done as described for Proteomics.

### Image processing

The images were processed and analyzed using Nikon's NIS-Elements version 5.02 platform. All sets were equally adjusted for all samples. Quantitative data are presented as the mean and standard error (SE). Sample sizes are indicated in the figure legends. For estimation of the ratio between contact and cytosolic Scribble (Fig. 6*C*), the peak GFP fluorescence intensities in cell–cell contacts or in cytosol in a middle confocal slice of cells expressing Scrib-GFP or ScribLK-GFP were used. For obtaining the apicobasal Par6 plots (Fig. 6*D*), confocal images (representing 50 μm × 50 μm squares of cell culture) that are representative of three independent experiments were used. Background subtraction was done using a constant value of 400 for all frames. All images were then converted to 2D “Volume View” in which the x-axis represented X-Y grids drawn with same density as Z-stacks (0.5 μm). The y-axis represented the z-axis of the original image. This measurement quantifies the number of grids (0.5 × 0.5 μm) within each confocal slice for which fluorescence exceeds the selected threshold (number of Par6 particles) and the fluorescence intensity of each of these grids (Par6 intensity). The result was transferred to Microsoft excel to plot the chart. For cell height analysis (Fig. 6*F*), the images were converted to two-dimensional volumetric view as before. Built-in Annotations and Measurements function was used for cell height measurement. A vertical straight line was drawn from the substrate to the top of the cell (marked by ZO1 staining). To quantify cells in the superficial layer (Fig. 6*G*), the whole image field was cropped into 10 μm equal slices and converted into two-dimensional volumetric view. A horizontal line was drawn dissecting the middle of the nuclei (DAPI stained) of the bottom layer of cells. The nuclei, which are not dissected by the line, were considered to be in the superficial layer. For TJ length analysis (Fig. 4), the entire ZO1-stained images were converted to Binary using NIH Fiji software. Detected TJ signal was skeletonized, and the branch length was quantified and summarized by using Skeleton analysis function. Quantification of the apical to cytosolic sLUR fluorescence ratio (Fig. 4*E*) was obtained using a total fluorescence of the 5 μm × 5 μm squares taken from the confocal slices of the apical membrane *versus* that of the identical squares taken from slices crossing the cytosol of the same cells. Quantitative data are presented as the mean and SD.

## Data availability

Raw mass spectrometry data are available in MassIVE Repository (https://massive.ucsd.edu/ProteoSAFe/static/massive.jsp) under the project ID MSV000088178 and in the PRIDE partner repository with the dataset identifier PXD028919 and 10.6019/PXD028919. All remaining data are contained within the article and supporting information.

## Supporting information

This article contains supporting information.

## References

[1] Bilder D. (2004). Epithelial polarity and proliferation control: Links from the Drosophila neoplastic tumor suppressors. Genes Dev..

[2] Tepass U. (2012). The apical polarity protein network in Drosophila epithelial cells: Regulation of polarity, junctions, morphogenesis, cell growth, and survival. Annu. Rev. Cell Dev. Biol..

[3] Rodriguez-Boulan E., Macara I.G. (2014). Organization and execution of the epithelial polarity programme. Nat. Rev. Mol. Cell Biol..

[4] Laprise P., Lau K.M., Harris K.P., Silva-Gagliardi N.F., Paul S.M., Beronja S., Beitel G.J., McGlade C.J., Tepass U. (2009). Yurt, Coracle, Neurexin IV and the Na(+),K(+)-ATPase form a novel group of epithelial polarity proteins. Nature.

[5] Chen J., Sayadian A., Lowe N., Lovegrove H.E., Johnston D. (2018). An alternative mode of epithelial polarity in the Drosophila midgut. PLoS Biol..

[6] Williams S.E., Ratliff L.A., Postiglione M.P., Knoblich J.A., Fuchs E. (2014). Par3-mInsc and Gαi3 cooperate to promote oriented epidermal cell divisions through LGN. Nat. Cell Biol..

[7] Legouis R., Jaulin-Bastard F., Schott S., Navarro C., Borg J.P., Labouesse M. (2003). Basolateral targeting by leucine-rich repeat domains in epithelial cells. EMBO Rep..

[8] Albertson R., Chabu C., Sheehan A., Doe C.Q. (2004). Scribble protein domain mapping reveals a multistep localization mechanism and domains necessary for establishing cortical polarity. J. Cell Sci..

[9] Zeitler J., Hsu C.P., Dionne H., Bilder D. (2004). Domains controlling cell polarity and proliferation in the Drosophila tumor suppressor Scribble. J. Cell Biol..

[10] Bonello T.T., Peifer M. (2019). Scribble: A master scaffold in polarity, adhesion, synaptogenesis, and proliferation. J. Cell Biol..

[11] Santoni M.J., Kashyap R., Camoin L., Borg J.P. (2020). The Scribble family in cancer: Twentieth anniversary. Oncogene.

[12] Kallay L.M., McNickle A., Brennwald P.J., Hubbard A.L., Braiterman L.T. (2006). Scribble associates with two polarity proteins, Lgl2 and Vangl2, via distinct molecular domains. J. Cell Biochem..

[13] Ivanov A.I., Young C., Den Beste K., Capaldo C.T., Humbert P.O., Brennwald P., Parkos C.A., Nusrat A. (2010). Tumor suppressor scribble regulates assembly of tight junctions in the intestinal epithelium. Am. J. Pathol..

[14] Choi J., Troyanovsky R.B., Indra I., Mitchell B.J., Troyanovsky S.M. (2019). Scribble, Erbin, and Lano redundantly regulate epithelial polarity and apical adhesion complex. J. Cell Biol..

[15] Ventura G., Moreira S., Barros-Carvalho A., Osswald M., Morais-de-Sá E. (2020). Lgl cortical dynamics are independent of binding to the Scrib-Dlg complex but require Dlg-dependent restriction of aPKC. Development.

[16] Khoury M.J., Bilder D. (2020). Distinct activities of Scrib module proteins organize epithelial polarity. Proc. Natl. Acad. Sci. U. S. A..

[17] Li X., Yang H., Liu J., Schmidt M.D., Gao T. (2011). Scribble-mediated membrane targeting of PHLPP1 is required for its negative regulation of Akt. EMBO Rep..

[18] Young L.C., Hartig N., Muñoz-Alegre M., Oses-Prieto J.A., Durdu S., Bender S., Vijayakumar V., Vietri Rudan M., Gewinner C., Henderson S., Jathoul A.P., Ghatrora R., Lythgoe M.F., Burlingame A.L., Rodriguez-Viciana P. (2013). An MRAS, SHOC2, and SCRIB complex coordinates ERK pathway activation with polarity and tumorigenic growth. Mol. Cell.

[19] Hilal M.L., Moreau M.M., Racca C., Pinheiro V.L., Piguel N.H., Santoni M.J., Dos Santos Carvalho S., Blanc J.M., Abada Y.K., Peyroutou R., Medina C., Doat H., Papouin T., Vuillard L., Borg J.P. (2017). Activity-dependent neuroplasticity induced by an enriched environment reverses cognitive deficits in Scribble deficient mouse. Cereb. Cortex.

[20] Nagasaka K., Seiki T., Yamashita A., Massimi P., Subbaiah V.K., Thomas M., Kranjec C., Kawana K., Nakagawa S., Yano T., Taketani Y., Fujii T., Kozuma S., Banks L. (2013). A novel interaction between hScrib and PP1γ downregulates ERK signaling and suppresses oncogene-induced cell transformation. PLoS One.

[21] Abe K., Takeichi M. (2008). EPLIN mediates linkage of the cadherin catenin complex to F-actin and stabilizes the circumferential actin belt. Proc. Natl. Acad. Sci. U. S. A..

[22] Kiss A., Troyanovsky R.B., Troyanovsky S.M. (2008). p120-catenin is a key component of the cadherin-gamma-secretase supercomplex. Mol. Biol. Cell.

[23] Nejsum L.N., Nelson W.J. (2007). A molecular mechanism directly linking E-cadherin adhesion to initiation of epithelial cell surface polarity. J. Cell Biol..

[24] Andreeva A., Lee J., Lohia M., Wu X., Macara I.G., Lu X. (2014). PTK7-Src signaling at epithelial cell contacts mediates spatial organization of actomyosin and planar cell polarity. Dev. Cell.

[25] Peradziryi H., Tolwinski N.S., Borchers A. (2012). The many roles of PTK7: A versatile regulator of cell-cell communication. Arch. Biochem. Biophys..

[26] Suzuki A., Ohno S. (2006). The PAR-aPKC system: Lessons in polarity. J. Cell Sci..

[27] Wettschureck N., Offermanns S. (2005). Mammalian G proteins and their cell type specific functions. Physiol. Rev..

[28] Silver J.T., Wirtz-Peitz F., Simões S., Pellikka M., Yan D., Binari R., Nishimura T., Li Y., Harris T.J.C., Perrimon N., Tepass U. (2019). Apical polarity proteins recruit the RhoGEF cysts to promote junctional myosin assembly. J. Cell Biol..

[29] Meiri D., Marshall C.B., Mokady D., LaRose J., Mullin M., Gingras A.C., Ikura M., Rottapel R. (2014). Mechanistic insight into GPCR-mediated activation of the microtubule-associated RhoA exchange factor GEF-H1. Nat. Commun..

[30] Merkley E.D., Rysavy S., Kahraman A., Hafen R.P., Daggett V., Adkins J.N. (2014). Distance restraints from crosslinking mass spectrometry: Mining a molecular dynamics simulation database to evaluate lysine-lysine distances. Protein Sci..

[31] Herzog F., Kahraman A., Boehringer D., Mak R., Bracher A., Walzthoeni T., Leitner A., Beck M., Hartl F.-U., Ban N., Malmström L., Aebersold R. (2012). Structural probing of a protein phosphatase 2A network by chemical crosslinking and mass spectrometry. Science.

[32] Kobe B., Kajava A.V. (2001). The leucine-rich repeat as a protein recognition motif. Curr. Opin. Struct. Biol..

[33] Bella J., Hindle K.L., McEwan P.A., Lovell S.C. (2008). The leucine-rich repeat structure. Cell. Mol. Life Sci..

[34] Keefe A.D., Wilson D.S., Seelig B., Szostak J.W. (2001). One-step purification of recombinant proteins using a nanomolar-affinity streptavidin-binding peptide, the SBP-Tag. Protein Expr. Purif..

[35] Laprise P., Tepass U. (2011). Novel insights into epithelial polarity proteins in Drosophila. Trends Cell Biol..

[36] Hendrickx A., Beullens M., Ceulemans H., Den Abt T., Van Eynde A., Nicolaescu E., Lesage B., Bollen M. (2009). Docking motif-guided mapping of the interactome of protein phosphatase-1. Chem. Biol..

[37] Choy M.S., Bolik-Coulon N., Archuleta T.L., Peti W., Page R. (2018). The structure of SDS22 provides insights into the mechanism of heterodimer formation with PP1. Acta Crystallogr. F Struct. Biol. Commun..

[38] Choy M.S., Moon T.M., Ravindran R., Bray J.A., Robinson L.C., Archuleta T.L., Shi W., Peti W., Tatchell K., Page R. (2019). SDS22 selectively recognizes and traps metal-deficient inactive PP1. Proc. Natl. Acad. Sci. U. S. A..

[39] Moreira S., Osswald M., Ventura G., Gonçalves M., Sunkel C.E., Morais-de-Sá E. (2019). PP1-mediated dephosphorylation of Lgl controls apical-basal polarity. Cell Rep..

[40] Rodrigues N.T., Lekomtsev S., Jananji S., Kriston-Vizi J., Hickson G.R., Baum B. (2015). Kinetochore-localized PP1-Sds22 couples chromosome segregation to polar relaxation. Nature.

[41] Grusche F.A., Hidalgo C., Fletcher G., Sung H.H., Sahai E., Thompson B.J. (2009). Sds22, a PP1 phosphatase regulatory subunit, regulates epithelial cell polarity and shape [Sds22 in epithelial morphology]. BMC Dev. Biol..

[42] Shao W., Wu J., Chen J., Lee D.M., Tishkina A., Harris T.J. (2010). A modifier screen for Bazooka/PAR-3 interacting genes in the Drosophila embryo epithelium. PLoS One.

[43] Indra I., Hong S., Troyanovsky R., Kormos B., Troyanovsky S. (2013). The adherens junction: A mosaic of cadherin and nectin clusters bundled by actin filaments. J. Invest. Dermatol..

