# Supplementary material for: Basolateral protein Scribble binds phosphatase PP1 to establish a signaling network maintaining apicobasal polarity
Source: J Biol Chem. 2021 Oct 8;297(5):101289. doi: 10.1016/j.jbc.2021.101289 (PMC8569552; doi:10.1016/j.jbc.2021.101289)
Supplement: Supplemental Table S1 [file mmc1.pdf]

#	GENE	Accession Number	Common protein name	LUR-517			LUR420			LUR-402			LUR-517 as base	LUR-420 %	LUR-402 %
				Spectra Count	Peptide Count	% of Coverage	Spectra Count	Peptide Count	% of Coverage	Spectra Count	Peptide Count	% of Coverage			
	SCRIB	SCRIB_HUMAN	Scribble	432	37	22.75%	414	29	19.80%	470	177	23.83%	100.00%	95.83%	0.00%
1	CTNNA1	ACTN1_HUMAN	Catenin alpha-1	87	35	55.28%	128	29	47.07%	96	54	47.00%	100.00%	147.13%	0.00%
2	LLGL2	L2GL2_HUMAN	Lethal(2) giant larvae protein homolog 2	75	34	44.10%	88	28	38.37%	21	14	14.87%	100.00%	117.33%	0.00%
3	PPP1CA	PP1A_HUMAN	Protein phosphatase PP1-alpha catalytic subunit (PP1A)	68	8	65.90%	68	18		7	7		100.00%	100.00%	0.00%
4	CTNND1	CTND1_HUMAN	p120 (ctn)	63	26	38.23%	68	24	36.23%	65	37	32.37%	100.00%	107.94%	0.00%
5	LLGL1	L2GL1_HUMAN	Lethal(2) giant larvae protein homolog 1	60	26	34.85%	58	20					100.00%	96.67%	0.00%
6	MYO1C	MYO1C_HUMAN	Unconventional myosin-Ic	59	25	31.65%	51	19	24.83%	89	52	40.40%	100.00%	86.44%	0.00%
7	CTNNB1	CTNB1_HUMAN	Beta-catenin	52	23	45.18%	67	19	39.50%	58	33	31.70%	100.00%	128.85%	0.00%
8	ATP1A1	AT1A1_HUMAN	Sodium pump subunit alpha-1	49	24	32.20%	50	21	27.47%	57	35	30.90%	100.00%	102.04%	0.00%
9	ITGB4	ITB4_HUMAN	Integrin beta-4	49	29	23.58%	52	23	19.30%	32	21	14.77%	100.00%	106.12%	0.00%
10	CNP	CN37_HUMAN	C-type natriuretic peptide	44	18	48.40%	40	14	39.13%	50	31	42.27%	100.00%	90.91%	0.00%
11	PKP3	PKP3_HUMAN	Plakophilin-3	32	15	27.93%	37	14	24.87%	51	32	32.87%	100.00%	115.63%	0.00%
12	GNAI3	GNAI3_HUMAN	Guanine nucleotide-binding protein G(i) subunit alpha	31	10	46.28%	32	10	37.10%	36	20	30.80%	100.00%	103.23%	0.00%
13	PPP1CB	PP1B_HUMAN	Protein phosphatase PP1-beta catalytic subunit (PP1B)	28	6	66.05%	12	5	54.03%	2	4	15.05%	100.00%	42.86%	0.00%
14	PPP1CC	PP1G_HUMAN	Protein phosphatase PP1-gamma catalytic subunit (PP1C)	27	17	71.00%	6	2		6	6		100.00%	22.22%	0.00%
15	GNB2	GBB2_HUMAN	G protein subunit beta-2	27	9	53.53%	26	7	43.63%	36	18	36.23%	100.00%	96.30%	0.00%
16	PTK7	PTK7_HUMAN	Protein-tyrosine kinase 7	27	16	22.98%	30	13	19.23%	20	14	11.83%	100.00%	111.11%	0.00%
17	MYO1D	MYO1D_HUMAN	Unconventional myosin-Id	26	16	21.80%	22	9	13.39%	10	6	5.89%	100.00%	84.62%	0.00%
18	EPB41L2	E41L2_HUMAN	Band 4.1-like protein 2	24	15	24.53%	33	14	21.57%	39	23	20.47%	100.00%	137.50%	0.00%
19	ZC3HAV1	ZCCHV_HUMAN	ARTD13	23	14	21.83%	21	10	18.10%	27	18	19.87%	100.00%	91.30%	0.00%
20	SLC3A2	4F2_HUMAN	4F2hc	23	11	25.03%	26	11	23.93%	27	18	24.47%	100.00%	113.04%	0.00%
21	EPB41L5	E41L5_HUMAN	Band 4.1-like protein 5	23	9	23.50%	28	11		2	2		100.00%	121.74%	0.00%
22	GNB1	GBB1_HUMAN	Guanine nucleotide-binding protein G(I)	20	9	53.90%	26	10	53.93%	16	11	34.70%	100.00%	130.00%	0.00%
23	DLG1	DLG1_HUMAN	Disks large homolog 1	19	12	22.74%	11	5	10.62%	16	10	11.33%	100.00%	57.89%	0.00%
24	MYO1B	MYO1B_HUMAN	Unconventional myosin-Ib	17	10	13.20%	22	8	11.15%	24	14	12.33%	100.00%	129.41%	0.00%
25	STXBP3	STXB3_HUMAN	Syntaxin-binding protein 3	17	9	21.35%	26	7	16.60%	16	9	12.17%	100.00%	152.94%	0.00%
26	ITGA6	ITA6_HUMAN	Integrin alpha-6	17	12	15.67%	15	8	11.35%	10	6	6.50%	100.00%	88.24%	0.00%
27	CDH1	CADH1_HUMAN	E-cadherin	16	8	15.57%	10	5	8.54%	22	13	14.60%	100.00%	62.50%	0.00%
28	CDC42	CDC42_HUMAN	CDC42	16	6	37.35%	17	4	28.97%	16	9	36.20%	100.00%	106.25%	0.00%
29	RAC1	RAC1_HUMAN	Ras-related C3 botulinum toxin substrate 1	14	6	40.33%	14	5	22.37%	18	11	36.50%	100.00%	100.00%	0.00%
30	DLG3	DLG3_HUMAN	Disks large homolog 3	14	7	13.48%	9	4		0	0		100.00%	64.29%	0.00%
31	RAB10	RAB10_HUMAN	Ras-related protein Rab-10	13	7	39.25%	14	4	43.00%	19	12	22.50%	100.00%	107.69%	0.00%
32	PLCB3	PLCB3_HUMAN	PLC-beta-3	13	8	11.51%	14	5	8.09%	11	10	8.78%	100.00%	107.69%	0.00%
33	GNAI2	GNAI2_HUMAN	Guanine nucleotide-binding protein G(i) subunit alpha-2	12	9	32.63%	8	4	32.47%	8	7	20.75%	100.00%	66.67%	0.00%
34	PKP2	PKP2_HUMAN	Plakophilin-2	11	6	11.25%	22	8	14.21%	19	11	12.52%	100.00%	200.00%	0.00%
35	EGFR	EGFR_HUMAN	Epidermal growth factor receptor	11	9	11.03%	11	4	5.54%	9	8	7.76%	100.00%	100.00%	0.00%
36	RAB8A	RAB8A_HUMAN	Ras-related protein Rab-8A	11	6	34.08%	20	7	34.97%	11	10	25.25%	100.00%	181.82%	0.00%
37	RHOG	RHOG_HUMAN	Rho-related GTP-binding protein RhoG	11	6	42.93%	16	6	37.70%	9	7	25.25%	100.00%	145.45%	0.00%
38	FAT1	FAT1_HUMAN	Protocadherin Fat 1	10	6	2.17%	16	8	3.42%	10	7	3.97%	100.00%	160.00%	0.00%
39	MARCKS	MRP_HUMAN	Mac-MARCKS	10	4	21.18%	3	2	11.55%	7	6	10.50%	100.00%	30.00%	0.00%
40	STXBP2	STXB2_HUMAN	Syntaxin-binding protein 2	10	5	16.70%	13	5	15.41%	6	6	10.20%	100.00%	130.00%	0.00%
41	GNA13	GNA13_HUMAN	G-protein subunit alpha-13	9	5	22.63%	19	7	28.03%	17	10	19.77%	100.00%	211.11%	0.00%
42	YES1	YES_HUMAN	Tyrosine-protein kinase Yes	9	6	15.58%	16	4	15.53%	10	9	14.35%	100.00%	177.78%	0.00%
43	RHOBTB3	RHBT3_HUMAN	Rho-related BTB domain-containing protein 3	9	6	15.28%	7	3	4.85%	13	8	13.93%	100.00%	77.78%	0.00%
44	ERBB2	ERBB2_HUMAN	Receptor tyrosine-protein kinase erbB-2	9	8	8.20%	8	5	6.59%	4	3	2.42%	100.00%	88.89%	0.00%
45	SEC61A1	S61A1_HUMAN	Sec61 alpha-1	9	2	8.48%	12	3	12.20%	14	9	11.75%	100.00%	133.33%	0.00%
46	EHBP1L1	EH1L1_HUMAN	EH domain-binding protein 1-like protein 1	9	6	7.02%	4	3		0	0		100.00%	44.44%	0.00%
47	JAK1	JAK1_HUMAN	Janus kinase 1	9	5	7.92%	20	6	7.98%	8	6	3.97%	100.00%	222.22%	0.00%
48	CASK	CSKP_HUMAN	Calcium/calmodulin-dependent serine protein kinase	8	5	7.17%	12	5	7.57%	5	5	7.39%	100.00%	150.00%	0.00%
49	ARHGEF2	ARHG2_HUMAN	Rho guanine nucleotide exchange factor 2 (GEF-H1)	7	3	5.71%	14	5	7.27%	38	23	20.73%	100.00%	200.00%	0.00%
50	DSG2	DSG2_HUMAN	Desmoglein-2	7	7	12.21%	15	8	12.11%	10	9	9.85%	100.00%	214.29%	0.00%
51	RRAS2	RRAS2_HUMAN	Ras-related protein R-Ras2	7	4	26.35%	5	4	26.50%	11	8	17.95%	100.00%	71.43%	0.00%
52	LYN	LYN_HUMAN	Tyrosine-protein kinase Lyn	7	6	20.75%	6	4	11.91%	5	4	6.09%	100.00%	85.71%	0.00%
54	RHOA	RHOA_HUMAN	Transforming protein RhoA	7	5	36.60%	10	3	27.63%	6	5	16.50%	100.00%	142.86%	0.00%
55	RAB35	RAB35_HUMAN	Ras-related protein Rab-35	7	5	36.08%	10	3	29.53%	8	7	19.90%	100.00%	142.86%	0.00%
56	NTPCR	NTPCR_HUMAN	Cancer-related nucleoside-triphosphatase	7	3	26.45%	10	4	30.87%	4	3	10.70%	100.00%	142.86%	0.00%
57	MPP7	MPP7_HUMAN	MAGUK p55 subfamily member 7	7	5	12.90%	9	3		0	0		100.00%	128.57%	0.00%
58	EHD4	EHD4_HUMAN	EH domain-containing protein 4	6	3	8.08%	7	4	11.04%	7	6	6.70%	100.00%	116.67%	0.00%
59	MET	MET_HUMAN	HGF receptor	6	3	3.96%	21	6	7.97%	8	5	4.88%	100.00%	350.00%	0.00%
60	RAB8B	RAB8B_HUMAN	Ras-related protein Rab-8B	6	3	30.95%	2	2	22.25%	6	5	22.50%	100.00%	33.33%	0.00%
61	ATP2B1	AT2B1_HUMAN	Plasma membrane calcium pump isoform 1	6	4	5.41%	20	7	10.35%	2	3	2.75%	100.00%	333.33%	0.00%
62	RAB21	RAB21_HUMAN	Ras-related protein Rab-21	6	3	19.00%	8	2	14.67%	4	4	9.85%	100.00%	133.33%	0.00%
63	MARK2	MARK2_HUMAN	Serine/threonine-protein kinase MARK2	6	3	6.54%	8	4	7.37%	7	5	6.83%	100.00%	133.33%	0.00%
64	PHLPP1	PHLPP1_HUMAN	PH Domain And Leucine Rich Repeat Protein Phosphatase 1	0	0	0.00%	0	0	0.00%	68	42	12.83%	100.00%		
65	LRRC40	LRC40_HUMAN	Leucine-rich repeat-containing protein 40	0	0	0.00%	2	2	5.40%	42	26	27.45%	100.00%		
