# Supplementary material for: Basolateral protein Scribble binds phosphatase PP1 to establish a signaling network maintaining apicobasal polarity
Source: J Biol Chem. 2021 Oct 8;297(5):101289. doi: 10.1016/j.jbc.2021.101289 (PMC8569552; doi:10.1016/j.jbc.2021.101289)
Supplement: Supplemental Table S2 [file mmc2.pdf]

	Genes Removed by P305L		Genes Removed by GFP Trap		Genes Removed by low abundance proteins	
	Gene	Spectra Count (mean)	Gene	Spectra Count (mean)	Gene	Spectra Count (mean)
1	HSP90AB1	330	FASN	153	MYOF	5
2	HSP90AA1	321	TUBB	144	S100A16	5
3	EEF2	173	TUBA1B	122	NCKAP1	5
4	ANXA2	97	PKM	118	CPNE3	5
5	HSPA8	97	FLNA	94	MARCKSL1	5
6	SUGT1	94	ACTG1	94	ACSL3	5
7	GCN1	83	EEF1A1	91	RAB5C	5
8	TLN1	62	KRT1	61	SH3BP4	5
9	CCT2	61	EIF4A1	54	OCLN	5
10	HSPH1	59	VCP	47	CUL3	5
11	PPIA	59	KRT10	43	EPHA2	5
12	STIP1	58	HSPA9	43	PPP2R5E	5
13	YWHAE	57	KRT9	42	RACK1	5
14	IPO5	44	YWHAZ	41	BCAM	4
15	VCL	44	UBA1	39	SLC9A3R1	4
16	TCP1	43	CLTC	38	CTNNA2	4
17	CCT5	41	CCT7	36	SDHA	4
18	CAND1	41	CCT6A	34	DPYSL2	4
19	CCT3	39	RPS3	34	RAB13	4
20	CACYBP	37	AHNAK	32	SLC7A5	4
21	DYNC1H1	36	ALDOA	30	PIP5K1A	4
22	CCT8	36	GAPDH	29	TBC1D10B	4
23	CCT7	36	SLC25A5	28	AFDN	4
24	HUWE1	35	EPRS	27	LIN7C	3
25	PPP2R1A	34	DDX3X	27	RHOF	3
26	IPO7	33	YWHAG	25	PLCD3	2
27	CCT4	31	CFL1	25	GIPC1	2
28	SLC25A6	29	HSPD1	23		
29	KPNB1	29	HSPA1A	23		
30	GART	28	UBA52	22		
31	XPO1	28	KRT2	21		
32	ACLY	28	EEF1G	19		
33	EIF5A	28	CSE1L	19		
34	SLC25A3	27	FLNB	17		
35	YWHAQ	27	PCBP1	16		
36	CFL1	25	PRDX1	15		
37	NUDC	24	RAN	15		
38	SLC25A13	24	PABPC1	15		
39	TARS	23	ENO1	13		
40	TUBB4B	21	RPS16	13		
41	JUP	21	RPL23	13		
42	YWHAB	21	PSMD2	12		
43	RUVBL1	20	RARS	12		
44	GFPT1	20	HNRNPK	12		
45	PAICS	20	EGFR	12		
46	ABCE1	20	PCBP2	12		
47	SLC25A11	20	IMPDH2	11		
48	DNAJA1	19	QARS	11		
49	VARS	19	PRDX2	11		
50	CLIC1	19	RAB8A	11		
51	TUBB3	19	FSCN1	11		
52	KRT8	19	DARS	10		
53	VPS35	19	PRKDC	9		
54	CAPN1	19	SERBP1	9		
55	DNAJA2	18	EIF4G1	9		
56	TOMM34	18	S100A10	9		
57	PDCD6IP	18	RPS20	8		
58	HSPA4	17	HSPA5	8		
59	TUFM	17	IARS	7		
60	RPS5	17	EIF3A	7		
61	RUVBL2	16	VDAC2	7		
62	PHGDH	16	MYH9	7		
63	EPS8L2	16	S100A6	7		
64	ARF4	16	COPA	7		
65	SLC25A1	16	XRCC5	6		
66	SLC25A10	16	HNRNPU	6		
67	ATAD3A	15	ATP2A2	5		
68	PPP5C	15	RPS2	5		
69	STAT1	15	COPB2	5		
70	GARS	15	NAP1L1	5		
71	GSTM3	15	TPM3	4		
72	YWHAH	15	XRCC6	4		
73	LASP1	15	RPL11	4		
74	CDC37	14	TKT	4		
75	TAGLN2	14	EEF1D	4		
76	PRDX6	14	LRPPRC	4		
77	TXNL1	14	TPI1	4		
78	MYO6	14	PDIA3	4		
79	ENO1	13	CTNNA2	4		
80	SPTAN1	13	PRSS1	4		
81	TUBB6	13	SFN	4		
82	KIF5B	13	SLC7A5	4		
83	PGD	13	ACTC1	3		
84	CYFIP1	13	NCL	3		
85	PSMD13	12	ACTN1	3		
86	PPP2R2A	12	NPM1	3		
87	AHCY	12	SND1	3		
88	BZW1	12	S100P	3		
89	DNAJC7	11	PTBP1	3		
90	SPTBN1	11	TPT1	3		
91	MMS19	11	RPS14	3		
92	RAB1A	11	UQCRC1	3		
93	FSCN1	11	EIF3C	3		
94	RPS27	11	KRT14	3		
95	CPOX	11	ANXA1	3		
96	PPP2CA	11	HRNR	3		
97	RPN1	10	TRIM28	2		
98	LRRC47	10	RPS11	2		
99	PSAT1	10	SHMT2	2		
100	DARS	10	RPS25	2		
101	TNPO1	10	SRP68	2		
102	RNH1	10	KRT5	2		
103	WDR1	10	EIF3L	2		
104	PPP3CA	10	CDCP1	2		
105	IPO9	9	SYNCRIP	1		
106	PPA1	9	CTTN	1		
107	TXN	9				
108	SKP1	9				
109	S100A10	9				
110	KPNA2	9				
111	TXNRD1	9				
112	RPS20	8				
113	GLRX3	8				
114	TMEM33	8				
115	RPL38	8				
116	HSPA5	8				
117	MARS	7				
118	PTGES3	7				
119	PSMD1	7				
120	OLA1	7				
121	USO1	7				
122	NPEPPS	7				
123	DDX19A	7				
124	PDIA6	6				
125	ATXN10	6				
126	CAD	6				
127	PCNA	6				
128	PSME1	6				
129	MTHFD1	6				
130	TPP2	6				
131	BZW2	6				
132	CAPZB	6				
133	TUBB4A	6				
134	MAP4	6				
135	ETF1	6				
136	UNC45A	6				
137	DDX6	6				
138	ADD1	6				
139	TUBA4A	6				
140	CSRP1	6				
141	SLC25A22	6				
142	G6PD	6				
143	SLC25A4	6				
144	AHSA1	6				
145	DENR	6				
146	INF2	6				
147	GDA	6				
148	USMG5	6				
149	RANBP1	6				
150	CSTB	6				
151	TPM4	6				
152	CTPS1	5				
153	PSME2	5				
154	TTC1	5				
155	ANKMY2	5				
156	ESYT1	5				
157	BID	5				
158	TES	5				
159	DNM1L	5				
160	ARFIP2	5				

**161**	EIF3B	5
**162**	MCM7	5
**163**	OXSR1	5
**164**	PSMD5	5
**165**	SLC16A1	5
**166**	HIGD2A	5
**167**	OCIAD2	5
**168**	TXNDC17	5
**169**	ECM29	5
**170**	MAPK1	5
**171**	ACP1	5
**172**	UBR4	4
**173**	PSMD12	4
**174**	VBP1	4
**175**	TTLL12	4
**176**	NUP93	4
**177**	CPNE1	4
**178**	DDX1	4
**179**	UGP2	4
**180**	CKAP5	4
**181**	GOT1	4
**182**	PFKL	4
**183**	SRP72	4
**184**	EIF4A2	4
**185**	HDLBP	4
**186**	PARK7	4
**187**	STUB1	4
**188**	SEC22B	4
**189**	CDC42BPB	4
**190**	UGDH	4
**191**	SMC2	4
**192**	NME1	4
**193**	TBCA	4
**194**	SLK	4
**195**	DNPEP	3
**196**	UBXN1	3
**197**	LDHA	3
**198**	PRPF8	3
**199**	PSMD3	3
**200**	TNS3	3
**201**	CAPNS1	3
**202**	ACTN1	3
**203**	MCM5	3
**204**	RRM1	3
**205**	EIF5	3
**206**	CARS	3
**207**	DNPEP	3
**208**	TUBA1C	3
**209**	EIF5B	3
**210**	COPS2	3
**211**	EIF3D	3
**212**	LRRFIP1	3
**213**	PRMT5	3
**214**	EIF2S2	3
**215**	USP5	3
**216**	CAMK2D	3
**217**	USP9X	3
**218**	RPL12	3
**219**	PFAS	3
**220**	UBE2N	3
**221**	PAFAH1B1	3
**222**	DPM1	3
**223**	CHORDC1	3
**224**	AP3B1	3
**225**	RNPEP	3
**226**	PFN1	3
**227**	EIF3F	3
**228**	PSMC5	3
**229**	TECR	3
**230**	GSPT1	3
**231**	VPS26A	3
**232**	PRPS2	3
**233**	GOT2	3
**234**	NDUFA4	3
**235**	UBE2L3	3
**236**	MCM3	2
**237**	IMMT	2
**238**	SGTA	2
**239**	AIMP2	2
**240**	ACADVL	2
**241**	PPM1B	2
**242**	SERPINH1	2
**243**	DBNL	2
**244**	ANXA11	2
**245**	PRDX5	2
**246**	SRPRA	2
**247**	DNAJB1	2
**248**	EML4	2
**249**	MOGS	2
**250**	GLMN	2
**251**	PFAS	2
**252**	RNPEP	2
**253**	HADHA	2
**254**	HLA-A	2
**255**	MCTS1	2
**256**	GPS1	2
**257**	RIC8A	2
**258**	SMC4	2
**259**	PFN2	2
**260**	DNM2	2
**261**	GANAB	2
**262**	HSPB1	2
**263**	HSP90AB4P	2
**264**	SEC24C	2
**265**	AGR2	2
**266**	BAG6	2
**267**	SNX2	2
**268**	NAPRT	2
**269**	NEK9	2
**270**	KPNA6	2
**271**	PCMT1	2
**272**	SEC23B	2
**273**	RTKN	2
**274**	EIF3G	2
**275**	MTCH2	2
**276**	CLIC4	2
**277**	EIF2S1	2
**278**	DDB1	2
**279**	GSR	2
**280**	ADSL	2
**281**	ATP6V1A	2
**282**	TNPO3	2
**283**	SYNCRIP	2
**284**	PSMD8	2
**285**	AARS	2
**286**	ROCK2	2
**287**	HDAC6	2
**288**	TBC1D4	2
**289**	DYNC1I2	2
**290**	STAT3	2
**291**	EIF4G2	2
**292**	UPF1	2
**293**	CSDE1	2
**294**	RPAP3	2
**295**	TOMM70	2
**296**	HSPA4L	2
**297**	H3F3A	2
**298**	DDX19B	2
**299**	GSR	2
**300**	TXNDC5	2
**301**	BAG2	2
**302**	CLUH	2
**303**	NADCD3	2
**304**	LARS	2
**305**	SPAG9	2
**306**	BLVRA	2
**307**	ATP6V1A	2
**308**	DSP	2
**309**	SLC25A12	2
**310**	RAP1GDS1	2
**311**	NCAPD2	2
**312**	TTI1	2
**313**	A2M	2
**314**	SERPINA1	2
**315**	ALB	2
**316**	HK2	2
**317**	MGST3	2
**318**	VPS51	2
**319**	HMGB1	2
**320**	AP3D1	2
